# High-Specificity Targeted Functional Profiling in Microbial Communities with ShortBRED

**DOI:** 10.1371/journal.pcbi.1004557

**Published:** 2015-12-18

**Authors:** James Kaminski, Molly K. Gibson, Eric A. Franzosa, Nicola Segata, Gautam Dantas, Curtis Huttenhower

**Affiliations:** 1 Department of Biostatistics, Harvard T.H. Chan School of Public Health, Boston, Massachusetts, United States of America; 2 Center for Genome Sciences and Systems Biology, Washington University School of Medicine, St. Louis, Missouri, United States of America; 3 Broad Institute, Cambridge, Massachusetts, United States of America; 4 Centre for Integrative Biology (CIBIO), University of Trento, Trento, Trentino, Italy; 5 Department of Pathology and Immunology, Washington University School of Medicine, St. Louis, Missouri, United States of America; 6 Department of Biomedical Engineering, Washington University, St. Louis, Missouri, United States of America; University of Washington, UNITED STATES

## Abstract

Profiling microbial community function from metagenomic sequencing data remains a computationally challenging problem. Mapping millions of DNA reads from such samples to reference protein databases requires long run-times, and short read lengths can result in spurious hits to unrelated proteins (loss of specificity). We developed ShortBRED (Short, Better Representative Extract Dataset) to address these challenges, facilitating fast, accurate functional profiling of metagenomic samples. ShortBRED consists of two components: (i) a method that reduces reference proteins of interest to short, highly representative amino acid sequences (“markers”) and (ii) a search step that maps reads to these markers to quantify the relative abundance of their associated proteins. After evaluating ShortBRED on synthetic data, we applied it to profile antibiotic resistance protein families in the gut microbiomes of individuals from the United States, China, Malawi, and Venezuela. Our results support antibiotic resistance as a core function in the human gut microbiome, with tetracycline-resistant ribosomal protection proteins and Class A beta-lactamases being the most widely distributed resistance mechanisms worldwide. ShortBRED markers are applicable to other homology-based search tasks, which we demonstrate here by identifying phylogenetic signatures of antibiotic resistance across more than 3,000 microbial isolate genomes. ShortBRED can be applied to profile a wide variety of protein families of interest; the software, source code, and documentation are available for download at http://huttenhower.sph.harvard.edu/shortbred

## Author Summary

High throughput DNA sequencing of the human microbiome presents a tremendous resource for researchers interested in studying microbial community functions such as antibiotic resistance. However, assigning DNA reads to protein families remains a challenging problem, as reads derived from a given protein-coding gene may map spuriously to homologous regions from unrelated proteins, which results in false positives. We addressed this problem with our method ShortBRED, which first identifies short peptide sequences (“markers”) that are highly representative for specific protein families, and then searches for these markers in metagenomic sequencing data to accurately detect and quantify protein families. In this work, we applied ShortBRED to profile antibiotic resistance in the healthy human microbiome of individuals worldwide and across bacterial genomes. ShortBRED can be similarly applied to profile many other protein families of interest.

## Introduction

Quantifying proteins of interest from metagenomic sequencing data in a fast and accurate manner is a central challenge in microbial community analysis. Whole metagenome shotgun (WMS) sequencing provides millions of short nucleotide sequences (often 100–250 bases long) from the DNA of organisms in a sample; we refer to these short DNA sequences as “reads.” A common approach to profiling protein families from these data involves (i) mapping reads to a database of reference protein sequences followed by (ii) interpreting the mapping results to estimate protein family relative abundance. This process is complicated by regions of local similarity in otherwise unrelated protein families: reads drawn from such regions will map non-specifically, which can result in false positive identifications (reduced specificity). Reducing the time spent on mapping reads is also an important task, as typical metagenomic sequencing depths and reference database sizes continue to grow rapidly.

Protein families are typically profiled in metagenomic sequencing data by one of three approaches: (i) mapping DNA reads to a database of nucleotide sequences [1], (ii) mapping translated DNA reads to a database of protein sequences [2], or (iii) assembling full-length genes from DNA reads *de novo* and then annotating them via comparison with reference databases [3]. Approaches (i) and (ii) rely on homology-based searches, as enabled by programs such as BLAST [4], USEARCH [5], and RAPSearch2 [6]. Methods such as MEGAN [7] and HUMAnN [2] can achieve very high sensitivity by mapping reads to large nucleotide and protein reference databases. However, this approach is vulnerable to false positives, as a read derived from a given protein-coding sequence may spuriously align to other genes as a result of local sequence homology, as mentioned above [8]. Moreover, searching nucleotide sequences against large, full-length protein reference databases comes at great computational cost, as search time is roughly proportional to database size and translated search is more computationally demanding than searching in nucleotide space. Assembly-based methods, while advantageous for identifying new genes, tend to underrepresent known, low-abundance genes, as more reads are required to assemble a gene than to identify it by homology-based search. Like search-based methods, assembly is also challenged by regions of local homology, which may lead to the construction of chimeric contigs.

False positive hits to regions of local homology can be mitigated by identifying and mapping against only unique substrings of protein sequences. This has the added benefit of reducing search time, as the unique portions of each database sequence constitute a smaller search space. One approach to this method involves identifying unique *k*-mers within bacterial protein sequences relative to a larger reference database [9]. While this approach makes progress toward increasing specificity and decreasing runtime, exact *k*-mer matching is not always biologically satisfying as it can fail to model common patterns in protein sequence evolution. For example, two peptide *k*-mers that differ by a single substitution between glutamic and aspartic acid (biochemically similar amino acids) are biologically similar, but would be scored as completely distinct by exact *k*-mer matching. Moreover, while *k*-mer approaches focus on matches to unique substrings of specific protein sequences, many metagenomics applications—particularly those involving poorly characterized microbial communities—benefit from alignment to more sequence-diverse protein families.

Here we present ShortBRED (Short, Better Representative Extract Dataset): a method for profiling protein family abundance in metagenomic data by first identifying short peptide markers that (i) are conserved within protein families and (ii) uniquely distinguish families from one another. ShortBRED achieves equivalent sensitivity, enhanced specificity, and enhanced speed relative to profiling strategies that map reads against full-length protein sequences. Unlike *k*-mer based profiling, ShortBRED relies on standard sequence homology-based methods to map reads to peptide markers, thus making it robust to common patterns in protein sequence evolution. By enabling faster, more accurate profiling of protein families in large metagenomes, ShortBRED allows researchers to better measure the prevalence and abundance of protein families of interest, and can lead to better understanding of biological phenomena. As proof-of-principle, we applied ShortBRED to profile antibiotic resistance (AR) families in both human microbiomes and bacterial isolate genomes, revealing new, population-specific, and phylogenetic trends in the distribution of this important class of proteins.

## Results

**Fig 1:**
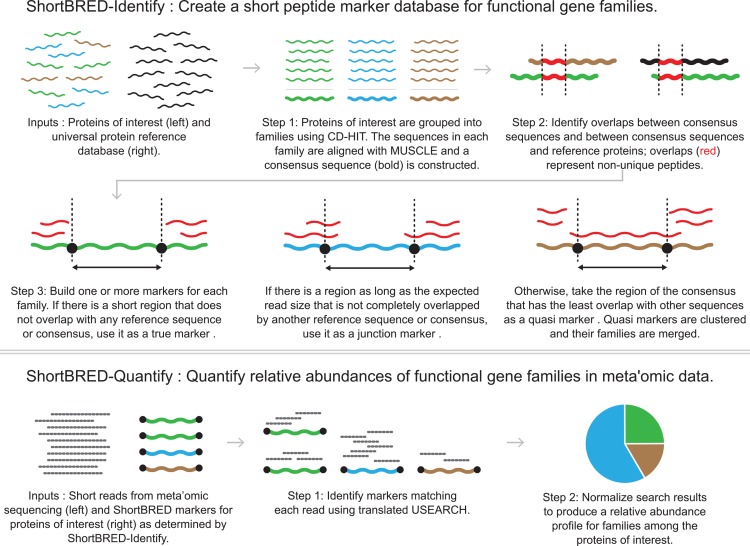
The ShortBRED algorithm. ShortBRED-Identify creates distinctive markers for protein families of interest. ShortBRED-Quantify maps nucleotides reads to markers and normalizes abundance.

We developed ShortBRED as a method to quickly and accurately quantify the relative abundance of protein families in WMS sequencing data. ShortBRED profiles protein family abundance in metagenomes by a two-step process: (i) *ShortBRED-Identify* isolates representative peptide sequences (markers) for the protein families, and (ii) *ShortBRED-Quantify* maps metagenomic reads against these markers to determine the relative abundance of their corresponding families (**Fig 1**). To evaluate ShortBRED, we measured its speed and accuracy in profiling synthetic metagenomes, and then tested its specificity by searching for yeast proteins in a synthetic bacterial metagenome. We next applied ShortBRED to profile AR genes in the gut microbiomes of healthy American [10], Chinese [11], and Venezuelan and Malawian [12] populations, as well as ~3,000 microbial isolate genomes.

### Profiling protein families in metagenomes using representative marker sequences

Current approaches to functional profiling of metagenomic samples often involve mapping reads to full-length protein sequences (e.g. centroid sequences of previously defined protein families). ShortBRED obtains higher speed and specificity relative to these approaches by reducing protein families to short, highly representative peptide sequences (markers), and then mapping reads against only those markers. To create the markers, *ShortBRED-Identify* uses two inputs: (i) a FASTA file of proteins-of-interest and (ii) a comprehensive reference database of additional protein sequences (provided as a FASTA file or preformatted BLAST database; **Fig 1**). *ShortBRED-Identify* first clusters the protein sequences of interest to identify protein families by global sequence homology, with each collapsed to form a single consensus sequence. Regions of a family’s consensus sequence that share strong, local sequence homology (“overlaps”) with proteins outside of the family are then penalized. Based on these overlaps, ShortBRED then isolates short peptide markers from the consensus that best represent the protein family. We classify these markers into three groups: *True Markers*, which do not overlap with the other protein families, *Junction Markers*, which overlap partially with the other protein families, and *Quasi Markers*, which are completely overlapped by another protein family.

The marker creation process is run once for a given set of proteins, resulting in a reusable and distributable marker database. *ShortBRED-Quantify* then (i) maps WMS sequencing reads against a given protein marker database using a translated search, (ii) counts high-quality hits, (iii) normalizes the counts based on marker length and sequencing depth, and (iv) produces a relative abundance profile of the protein families of interest represented by the marker database (**Fig 1**). Creating a highly specific marker sequence database has two major advantages: (i) searches against this database are more accurate, as the exclusion of non-specific (overlap) regions reduces false positive hits, and (ii) searches proceed more quickly, as the search space is considerably reduced relative to the full database.

### ShortBRED achieves better specificity than mapping reads to centroids

We constructed synthetic datasets to train ShortBRED’s default parameter settings and validate its performance. For one set of AR protein families (ARDB [13]) and one set of virulence factor protein families (VFDB [14]), we created three synthetic bacterial metagenomes spiked with the proteins of interest at 5%, 10%, and 25% relative abundance. We first tuned ShortBRED’s ability to correctly call the presence and absence of protein families in the 5%-spiked metagenomes by varying the initial protein clustering identity (80%, 85%, 90%, 95%, and 100%) and minimum allowed marker length (8, 10, 12, 15, 18, 20, 22, 25, and 30 amino acids; **S1–S8 Figs**). We first restricted the allowed parameter space to those combinations yielding a specificity of at least 99% in our initial evaluation. From among these combinations, we selected a clustering identity of 85% and minimum marker length of 8 amino acids as ShortBRED’s defaults as they gave the best sensitivity performance on the ARDB-spiked metagenome (there was little variation in performance on the VFDB-spiked metagenome). These parameter settings were used for the remaining analyses in this work; they can be easily tuned with command-line arguments for other applications.

To further validate our parameter settings and ShortBRED’s performance, we generated markers for the ARDB and VFDB protein families based on the optimal settings described above (**Table 1**). We then used these markers to profile six synthetic metagenomes, including the 10%- and 25%-spiked metagenomes that were not used in the training process. We compared ShortBRED to an alternative profiling strategy in which reads were mapped directly to the centroid sequences of protein families. Centroids were obtained by clustering the proteins of interest at 85% identity; during the quantification stage, any matches to centroids with length ≥30 amino acids and ≥95% identity were considered valid hits.

**Table 1:** Characteristics of ShortBRED markers used to profile synthetic metagenomes.

	ARDB	VFDB
**Families after initial clustering**	618	2,089
Families with true markers	594	2,041
Families without true markers	24	48
**Total markers**	2,886	7,869
True markers	2,845	7,730
Junction markers	37	139
Quasi markers	4	0

Legend: This table lists the number of protein families and maker types present in the ARDB and VFDB markers created by ShortBRED-Identify for profiling synthetic metagenomes.

**Fig 2:**
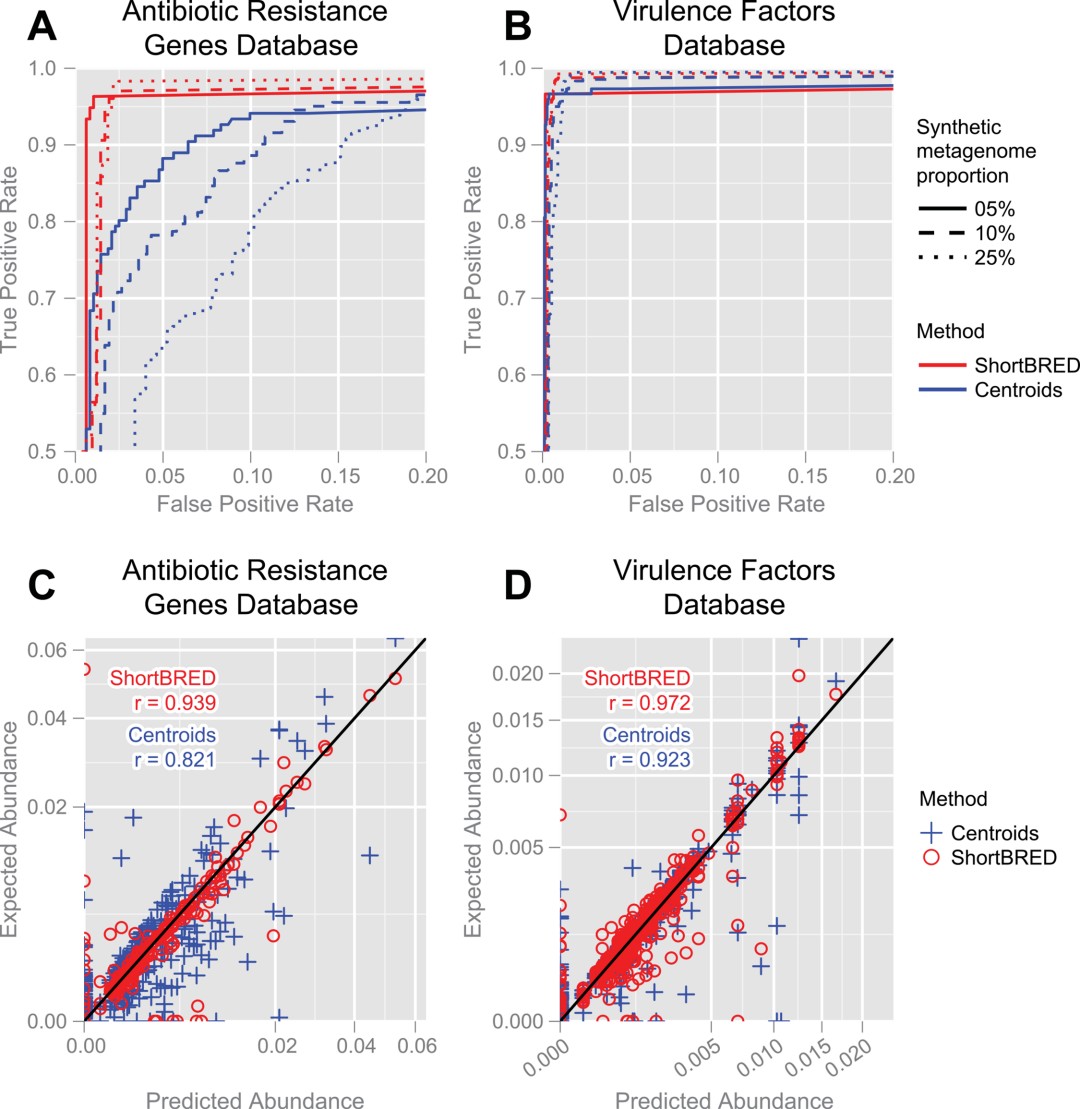
Accuracy of ShortBRED and centroid-based profiling within synthetic metagenomes. (**A**, **B**) ROC curves report the sensitivity and specificity (in terms of TPR and FPR) of the two methods for correctly identifying the presence and absence of protein families of interest in six synthetic metagenomes, spiked with 5%, 10%, and 25% of their material from the ARDB (panel A) and VFDB (panel B). (**C**, **D**) Scatterplots of protein family “predicted from mapping”, the abundance values calculated by ShortBRED and the centroids, versus “expected from gold standard”, the abundance values of the protein families in the 10% synthetic metagenome.

An ideal search methodology will correctly identify all protein families present in a metagenome (true positive rate, TPR, equal to 1) and will not erroneously identify any protein families absent from the metagenome (false positive rate, FPR, equal to 0). As we intensify the criteria for calling a family as present (e.g. requiring a higher normalized count for the family), TPR and FPR will both decrease: a tradeoff we quantify using a receiver operating characteristic (ROC) curve (**Fig 2A and 2B**; **S1 Table**). Notably, even treating a single hit to a ShortBRED marker as evidence of the corresponding protein family’s presence resulted in exceptional sensitivity with very low false positives (<5%). As we increased the number of protein families present and the share they comprised of the metagenome, ShortBRED achieved TPR and FPR values comparable to or exceeding those of the centroids method (**S1 Table**). Since greater spike-in percentages provided more opportunities for centroids from one cluster to match to reads from another cluster by local homology, the centroids method performed well for the 5%-spiked metagenomes but experienced a substantial drop in specificity at similar levels of sensitivity to ShortBRED in analyses of the 10%- and 25%-spiked metagenomes.

Beyond correctly calling protein family presence and absence, an ideal search strategy will be able to accurately quantify the relative abundances of these families in a metagenome, which may vary over several orders of magnitude. Using Spearman’s correlation to compare known and predicted relative abundances, ShortBRED outperformed the centroid-based method in the six Illumina metagenomes (median *r* = 0.95 versus 0.82; **S1 Table**). The weaker performance of the centroid-based method was due in part to a larger fraction of false positive detection events (defined to have 0 expected abundance; **Fig 2C and 2D**). We additionally performed a more challenging evaluation on sequences with either 3% or 5% amino acid substitutions, retaining specificity >0.95 and >0.98 respectively, and sensitivities >0.88 and >0.79 (**S1 Table**). This is in contrast to centroid matching on the same datasets, which achieved minimum specificities of only 0.80 and 0.77, respectively. Thus, ShortBRED’s increased specificity not only provides a more accurate qualitative profile of protein family presence and absence, but also contributes to more accurate quantitative profiling. As an additional evaluation of specificity, we applied ShortBRED and the centroid-based profiling method to search for yeast proteins in a synthetic bacterial metagenome. Given that yeast and bacteria are extremely distantly diverged [15], homology between a short, bacterial DNA sequence and a yeast protein is likely to have resulted from chance. ShortBRED did not identify any false positive hits to yeast proteins among the bacterial DNA reads, while the centroid-based method produced fifteen high-identity and long-length hits (**S2 Table**). The centroid-based profiling method offered some advantages over ShortBRED when working with shallow sequencing data (e.g. as derived from older 454 sequencing experiments), wherein reads are less likely to have been sampled from marker regions (**S9 Fig**). However, this limitation vanishes when working with typical modern sequencing depths, while the drawbacks of the centroid-based approach will only grow as typical depths continue to increase.

### ShortBRED is faster than centroid-based profiling

**Fig 3:**
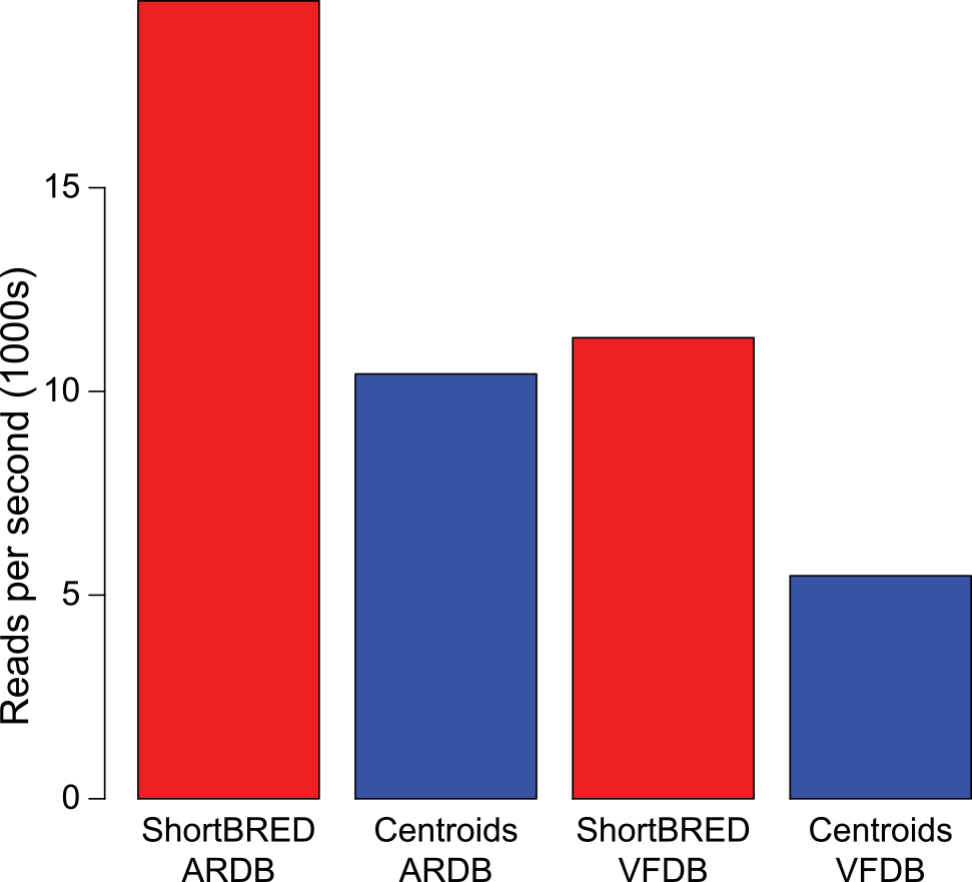
Speed of execution: ShortBRED versus centroid-based profiling. Results are based on time used by USEARCH in ShortBRED-Quantify.

In addition to increasing the accuracy of metagenomic search, ShortBRED’s focus on a reduced sequence database (the markers) results in considerably shorter run-times relative to searching against full-length centroids (**Fig 3**). Focusing on metagenomes spiked with proteins from ARDB, we were able to process ~10,400 reads/sec (on average) by mapping against full-length ARDB centroid sequences, while ShortBRED processed ~19,600 reads/sec using the previously generated ARDB marker sequences (a 1.9x increase in speed). For the VFDB-spiked metagenomes, we were able to map ~5,400 reads/sec against centroid sequences, while ShortBRED processed ~11,300 reads/sec (a 2.1x increase in speed). All mapping experiments were carried out on the same computer hardware using 5 CPU cores and the same underlying mapping program (USEARCH); hence, ShortBRED’s increased speed can be attributed to the reduced size of the marker database. For a modern metagenomics study producing 100s of millions of reads for 100s of samples, this speedup corresponds to savings of 100s of CPU-hours of compute time.

### Antibiotic resistance in the human gut microbiome worldwide

**Fig 4:**
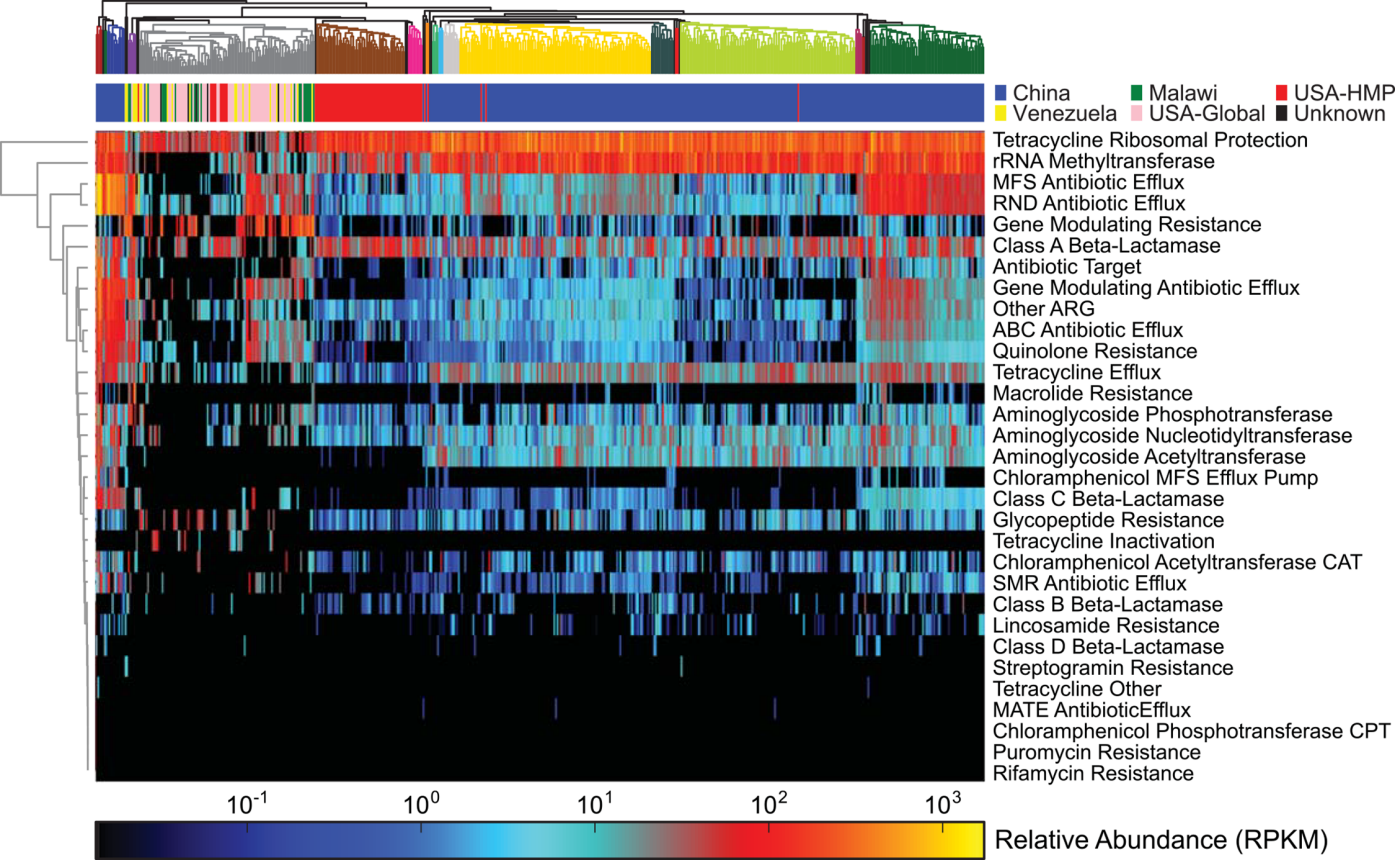
Antibiotic resistance in the human gut microbiome. RPKM values produced by ShortBRED for antibiotic resistance protein families, summed by class of resistance. Samples in the USA-Global, Venezuela, and Malawi cohorts were profiled by mapping reads to centroids due to their lower sequencing depth. Marker information is listed in **Table 2**. Samples (columns) were clustered according to Canberra distance and antibiotic resistance families (rows) were clustered according to Euclidean distance.

We leveraged the improved specificity of ShortBRED to accurately quantify antibiotic resistance (AR) worldwide in the human gut microbiomes of 552 individuals from the United States [10, 12], China [11], Malawi, and Venezuela [12] (**Fig 4**and **S3 Table**). We identified centroid sequences (appropriate for the more shallow 454 sequencing in [12]) and ShortBRED markers (for Illumina sequences from 10–12) for 849 AR protein families derived from the ARDB and independent curation [16]. These families were further grouped into broader classes such as such as “Class A beta-lactamase” and “quinolone resistance.” 107 microbiome samples based on older 454 sequencing methods were mapped to centroid sequences to avoid loss of sensitivity from low sequencing depths; all other samples were profiled with ShortBRED (see Methods).

Our results support AR as a core function in the human gut microbiome, with every individual gut microbiome containing at least one AR determinant (**Fig 4**). As previously observed [17, 18], resistance to the tetracyclines was the most widespread AR function in the human gut microbiome, with at least one tetracycline resistance mechanism being identified in 99% of individuals across all three studies' global populations (97% ribosomal protection; 87% efflux; 3% inactivation). In addition, Class A beta-lactamases were identified in 90% of individuals and were widespread throughout all populations, with CfxA and CblA the most common variants (as represented by families P30898 at 68.5% prevalence and P30899 at 60.1% prevalence; see **S4 Table**). Based on the diversity of participant ages present particularly in the Yatsunenko et al study, these prevalent AR families appear early in life and appear cross-sectionally across a wide range of subject demographics.

Consistent with previous findings [19], this global distribution of AR determinants in the human gut microbiome appears to be driven by the underlying bacterial phylogenetic profile. For example, while Class A beta-lactamases are known to be the most diverse and widely disseminated class of beta-lactamase genes [20], the most abundant variants (CfxA and CblA) have been previously shown to be specific to *Bacteroides* species [21, 22]. Hence, enrichment for these families may be a direct marker for the presence of specific bacterial clades in the gut microbiota rather than a response to selective pressures of individual-specific antibiotic use. The relationship between microbiome-specific AR and phylogenetic profiles are addressed in greater detail in subsequent sections.

In addition to the universal AR trends described above, ShortBRED revealed several consistent differences in AR profiles between global populations. For example, Chinese individuals were particularly enriched for a number of AR factors: quinolone resistance, aminoglycoside acetyltransferases, and genes modulating antibiotic efflux. Among these individuals, the two most prevalent quinolone resistance families (variants of fluoroquinolone-resistant DNA topoisomerases) were found in 78% and 29% of individuals, the most prevalent aminoglycoside acetyltransferase (YP_002559372) was found in 99.7% of individuals, and the next-most-prevalent (P13246) followed at 19.3% of individuals. Four gene-modulating antibiotic efflux families (phoQ_1, soxR_5, marA_1, and baeR_2) had individual prevalence values exceeding 58% (**S4 Table**). In addition, while many AR genes were discretely strongly present or absent within the Chinese cohort (**Fig 4**), their gut resistomes were differentiated into four clear clusters based largely on the abundance of antibiotic efflux pumps, including major facilitator superfamily (MFS) antibiotic efflux, resistance/nodulation/cell division (RND) antibiotic efflux, and small multidrug resistance (SMR) antibiotic efflux pumps. Many multidrug antibiotic efflux pumps are chromosomally encoded and highly conserved across all members of a given bacterial species [23], further suggesting that observed AR distribution patterns are driven by underlying community membership and phylogeny.

In comparison with the Chinese cohort, gut microbiome samples from the American (HMP) cohort were much more homogeneous. This difference was likely influenced by the greater diversity in membership among the Chinese cohort, which contained individuals with and without type II diabetes and a wide range of ages (13–86). In comparison, the HMP cohort consisted solely of young (ages 18–40), healthy individuals. Differences between the cohorts may also reflect variation in the sampling and sequencing protocols used by their corresponding studies (in addition to real biological variation). American individuals were characterized by increased abundance of four protein families within the Class A beta-lactamases (CfxA_11, AAA22905, P30898, and P30899; **S4 Table**). Conversely, these individuals were depleted for aminoglycosides and acetyltranferases. These observed differences between the American and Chinese cohorts stress that, while AR is (at a high level) core to the global human gut microbiome, variation emerges in specific resistances present in individual populations.

### Connecting antibiotic resistance to phylogeny

In order to understand and control the spread of AR, it is necessary to characterize the connections between AR determinants, source genomes and their phylogeny, and the relative propensity of horizontal gene transfer (HGT). In addition to their usefulness in metagenomics profiling, ShortBRED markers can aid in this goal by providing highly specific signatures of AR protein families for microbial genome annotation. We used ShortBRED to profile the 849 AR protein families introduced above across 3,305 phylogenetically diverse microbial isolate genomes [24] (see Methods).

**Fig 5:**
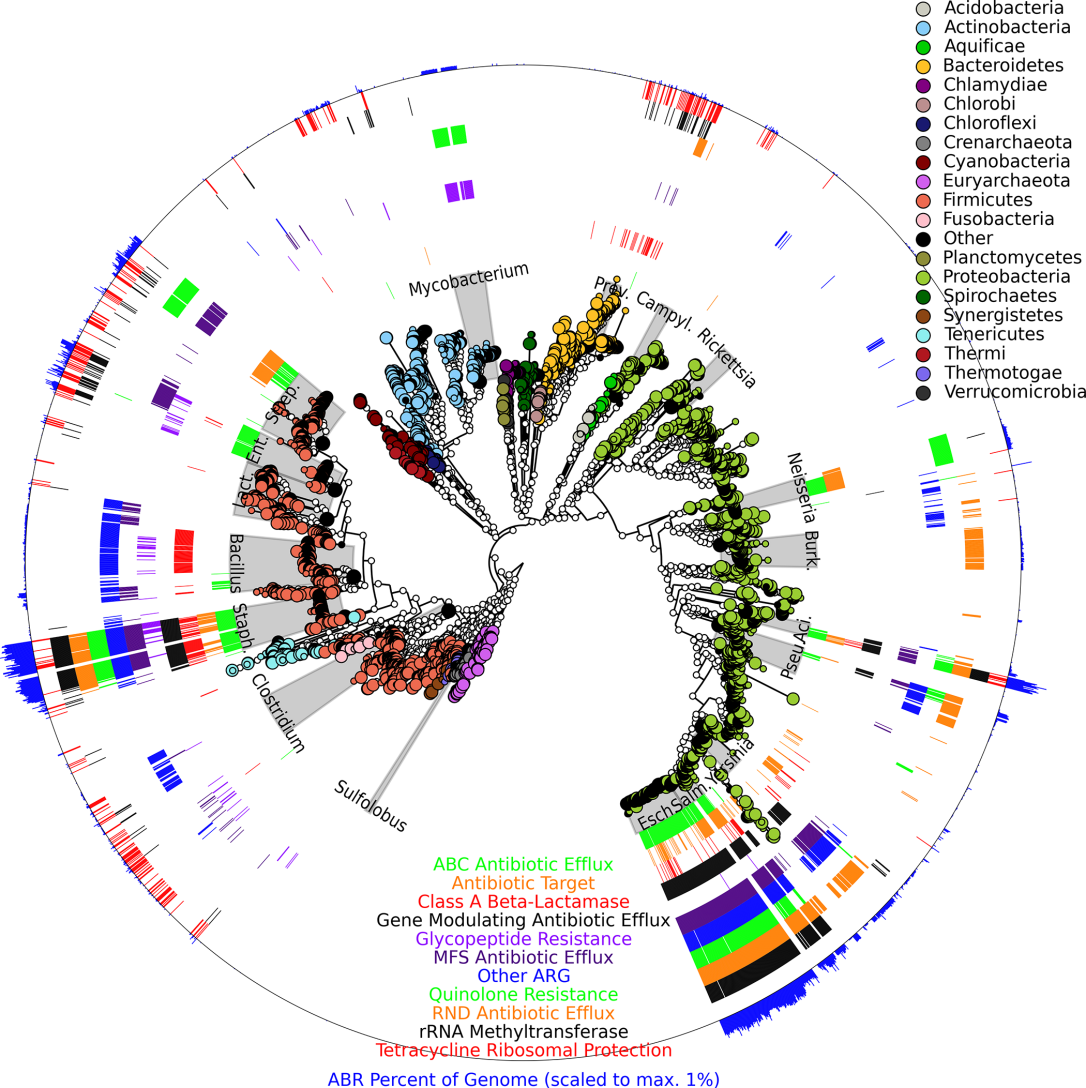
Prevalence of antibiotic resistance across bacterial isolate genomes. Phylogenetic tree of bacterial genomes from IMG [24] overlaid with presence/absence of ShortBRED antibiotic resistance protein families. The outermost ring indicates the share of genes in each species’ genome that mapped to any of the AR protein families. This figure was produced using GraPhlAn [27].

Over 40% of microbial isolate genomes surveyed encoded at least one AR determinant, with significant enrichments among particular genera (**Fig 5**). For example, *Escherichia* and *Salmonella* are closely related bacterial genera that contain many human pathogens [25, 26]; both were highly enriched for AR determinants. Specifically, all *Escherichia* and *Salmonella* encoded at least one AR class, with an average of 20.3 AR genes for *Escherichia* and 11.2 AR genes for *Salmonella* (**S5 Table**). In addition, while these two genera shared many similar AR determinants, they appear to resist beta-lactam antibiotics using largely orthogonal mechanisms: 94.6% of *Escherichia* genomes were enriched for Class C beta-lactamases and were completely depleted of Class B beta-lactamases, while *Salmonella* showed the opposite trend (6.5% of genomes encoded Class C resistance, while all genomes encoded Class B resistance, **S10 Fig, S5 Table**). While these examples illustrate cases of strong coupling between AR determinants and particular genera, this was not always the case. For example, glycopeptide resistance was highly variable within the genus *Enterococcus*, with ~1/3 of isolate genomes possessing the function while the remaining 2/3 lacked it.

Our observations further suggested that AR functions could be subdivided into two categories of phylogenetic distribution: (i) functions that are clade-specific, i.e. highly conserved across all members of a bacterial clade, and (ii) functions that are broadly distributed across the phylogenetic tree. Both distribution patterns were observed among abundant AR classes in the human gut microbiome (**Fig 5**and **S5 Table**). For example, multi-drug antibiotic efflux pumps and rRNA methyltransferases showed strong signatures of clade-specific enrichment among the *Staphylococcus*, *Escherichia*, *Salmonella*, and *Yersinia* genera. Functions that are tightly linked to particular clades are notable in that their presence and abundance can be inferred from profiles of community composition alone, including profiles based on lower-resolution amplicon sequencing [28]. Conversely, tetracycline ribosomal protection determinants were widely dispersed across the phylogenetic tree—a pattern more consistent with recent spread by mechanisms such as HGT [1]. The presence and abundance of functions in this category would be difficult to infer from community profiling and are best quantified directly from a metagenome—a process facilitated by ShortBRED.

### Predicting antibiotic resistance profiles from community composition

The previous section stressed that, while some AR functions can be accurately quantified based on microbial community composition, broadly distributed functions pose a greater challenge. To further explore this idea, we compared observed and predicted AR profiles for 82 gut metagenomes from HMP individuals. We predicted the AR profile for a given gut metagenome by first quantifying the sample’s microbial community composition with MetaPhlAn [29]. This step resulted in a vector of relative abundance measurements for species present in the sample (in RPKM units). Then, using the ShortBRED based-annotations of AR functions in bacterial genomes described above, we computed the abundance of each AR function in the sample by multiplication. For example, if species A had a relative abundance of 5 RPKM and contained 1 copy of AR protein X, while species B had a relative abundance of 10 RPKM and contained 2 copies of AR protein X, then the total abundance of AR protein X in the metagenome was predicted to be:

1(5RPKM)+2(10RPKM)=25RPKM.

This procedure was repeated for all samples and AR functions.

At the level of individual AR gene families, ShortBRED and the predicted profiles co-detected 63 families, 57 were detected by ShortBRED but never observed in the predicted profiles, and 14 were predicted to be present but never confirmed by ShortBRED. Among the co-detected families, the average quantitative agreement between the ShortBRED and predicted profiles (as measured by Spearman’s correlation) was 0.43. When gene families were grouped into broader AR classes, 17 were co-detected, 5 were found only by ShortBRED, and 1 was predicted to occur but not confirmed by ShortBRED. Average quantitative agreement for the 17 co-detected classes was 0.33 (Spearman’s correlation). The AR classes most under-represented by the community composition-based predictions were tetracycline ribosomal protection, Class A beta-lactamase, rRNA methyltransferase, MFS antibiotic efflux, and RND antibiotic efflux (**S6 Table**). Notably, tetracycline resistance was also among the most broadly-distributed AR classes.

In addition, individual-specific ShortBRED-based versus predicted AR profiles showed poor quantitative agreement (average Spearman correlation = 0.53). There are a number of reasons why the two profiles would agree poorly on an individual basis or for particular AR families. While ShortBRED is able to profile AR gene abundance in cases where the genes are present in uncharacterized genomes, the taxonomic profile method is limited to species with known isolate genomes. Hence, predicting AR content from taxonomic composition will tend to underestimate AR content, and explains why ShortBRED detects several families that the predictive method does not. In instances where multiple isolate genomes were available for a species detected in a sample, the species’ contributions were based on the median gene copy number for each AR family across its isolate genomes. If the sample isolate contained fewer copies of an AR gene than the median estimate, then we would tend to overestimate its abundance; conversely, if the sample isolate contained more copies of an AR gene than the median estimate, then we would tend to underestimate its abundance (both serving to weaken signal-to-noise ratio among the predictions). For these reasons, directly profiling AR content in a metagenome is preferable to predicting functional content from community composition. ShortBRED offers a means to profile AR content and other protein families in a fast, highly specific manner.

## Discussion

In this work, we have presented and validated ShortBRED: a tool to build short peptide markers for protein families and then apply them to profile protein family content in a metagenomic sequencing sample. We demonstrated that ShortBRED is both faster and more accurate than the common approach of mapping reads to full-length protein family centroid sequences. ShortBRED is extensible to a diverse collection of functional profiling tasks. The most straightforward of these was demonstrated in our profiling of antibiotic resistance in human gut metagenomes, which we discuss further below. Although this example was based on DNA sequence data, ShortBRED’s markers are also applicable for profiling microbial community RNA-Seq data (metatranscriptomics), which reveals the relative functional activity of protein families in a community. In addition to profiling meta’omic sequencing data, ShortBRED’s markers have proven useful for identifying protein families of interest in microbial isolate genomes, as the markers’ small sizes and highly representative sequences facilitate rapid, unambiguous gene annotation. The functional profiles produced in these applications are amenable to a variety of downstream analysis methods, including comparing functional composition in case versus control samples or monitoring temporal variation in functional composition or activity from longitudinal samples.

Mapping metagenomic reads to protein families of interest is an example of a search problem in which new queries (samples) arise more frequently than changes to the database (proteins of interest). In such cases, it is desirable to pre-process the database in order to accelerate downstream search. ShortBRED accomplishes this by reducing large numbers of protein sequences first to clusters of related proteins (families) and then to representative peptide markers. Searching a new metagenomic sample against these marker sequences represents a considerable savings in computation relative to searching against the full database. ShortBRED’s pre-processing steps, while not computationally trivial, can reduce a collection of ~1,000 protein families to identifying markers in a matter of hours on typical desktop or server hardware (i.e. taking advantage of multiple CPU cores for parallelization, but not requiring special high-memory or accelerated file I/O configurations). The bottleneck in this process is the BLAST-based search of the proteins of interest against the universal protein reference database. In the future, it may be possible to further accelerate pre-processing steps by incorporating an alternative program for protein homology search, provided that it meets or exceeds BLAST’s sensitivity. In the same vein, downstream performance mapping reads to markers depends largely on the speed of ShortBRED’s chosen translated search tool (currently USEARCH), which could also be replaced or supplemented by future alternatives.

In our evaluations, the vast majority of protein families could be identified by one or more unique amino acid subsequences (True Markers). Although these sequences are used here for protein family identification and quantification, they are themselves interesting targets for investigation. For example, the conservation of these sequences within a family may indicate the presence of a functionally relevant domain, peptide recognition motif, or enzyme active site. The small minority of protein families that lacked unique identifying subsequences are also worthy of consideration (**Tables 1**and **2**). In such cases, ShortBRED constructs a Quasi Marker to represent the family: i.e. the amino acid sub-sequence which, while not unique to the family, is found in a minimal number of other families. Users may wish to exclude Quasi Markers (and their associated families) in their analyses to increase specificity. That said, Quasi Markers were always included in the analyses reported here and were found to compromise specificity only slightly (far less than the centroid-based approach; **Fig 2**and **Table 2**). In the future, an expectation maximization (EM) step could be incorporated in ShortBRED-Quantify to improve the accuracy of protein family quantification when mapping reads to ambiguous Quasi-Markers

**Table 2:** Characteristics of ShortBRED markers used to profile metagenomes and bacterial genomes.

	HMP	T2D	T2D_Short	Yatsunenko	Bacterial Genomes
**Parameter settings**					
Clustering ID	8	8	8	8	8
Minimum marker length	85%	85%	85%	85%	85%
Average read BP	101	90	75	450	100
Min trusted BP	90	81	68	200	30
QM length	30	27	22	66	33
**Statistics**					
Families after initial clustering	849	849	849	849	849
Families with true markers	820	820	820	**	820
Families without true markers	29	29	29	**	29
**Total markers**	4132	4135	4142	**	4132
True markers	4078	4078	4078	**	4078
Junction markers	48	50	61	**	48
Quasi markers	6	7	3	**	6
**Dataset profiled**					
Samples profiled	82	272	91	107	3305

Legend: This table lists characteristics of the markers used to profile the metagenomes and bacterial genomes. Each metagenome from the Chinese cohort was profiled with one of two sets of markers (T2D and T2D_Short) corresponding to the two different read sizes used in the dataset (90 and 75 bp) None of the families without True Markers were combined in the second round of clustering.

** Centroids were used to profile the Yatsunenko dataset.

We demonstrated ShortBRED’s utility by generating and applying AR gene markers to profile AR gene content in 552 human gut metagenomes and 3,305 bacterial isolate genomes. AR determinants in pathogens are increasingly compromising infectious disease treatment due to their acquisition from commensal or environmental bacteria [30, 31]. The human gut microbiome serves as a transferable reservoir of AR readily available to human pathogens [32], leading to an increased focus on characterization and quantification of AR genes in large metagenomic studies [17, 18, 33]. However, accurate quantification of AR genes using short reads is challenging: AR determinants are often originally genes with diverse native functions repurposed through mutation or expression modulation to provide AR [34], therefore sharing large sequence similarity to genes with no AR function. For example, when particular RND efflux pumps (such as CmeABC, AcrB, and Mex) highly expressed, they are capable of exporting multiple antibiotics [35]. However, members of the RND efflux pump superfamily also serve important functions as transporters of proteins required for nodulation and cell division and, while they do not always demonstrate inherent AR activity, they share high sequence similarity with proteins shown to serve resistance functions. As a result, previous attempts to profile antibiotic resistance in human gut metagenomes by mapping short reads to full-length protein sequences may have been compromised by spurious mapping events.

Our ShortBRED-based profiles avoided this complication by using only the most information-rich portions of AR genes for identification and quantification of AR in microbial communities and isolate genomes. Notably, our results agree with several of the major findings from previous profiling attempts, specifically (i) high relative abundance of AR genes in the gut microbiota of Chinese individuals compared to individuals from other countries as well as (ii) ubiquity of tetracycline resistance worldwide [17, 18]. Hence, we can be confident that these results are not the result of spurious mapping to full-length protein sequences. ShortBRED demonstrated increased sensitivity for identification of additional classes of AR genes, including resistance to the quinolone class of antibiotics. The application of ShortBRED to the identification and quantification of AR genes in microbial communities addresses a significant challenge in the computational investigation of AR using high-throughput sequencing technology. In addition, just as ShortBRED markers enabled confident differentiation between closely-related AR and non-AR proteins in metagenomes, the same advantage applied to annotating full-length protein sequences in bacterial isolate genomes. Indeed, we used this method to dissect phylogenetic properties of the AR families under study, revealing distinct patterns of clade-specific versus broad distribution. In the future, the same technique could be applied to quickly and accurately determine AR gene content in a newly-sequenced bacterial strain—an application with relevance to infectious disease management.

To facilitate such applications, the antibiotic resistance markers produced here are available for download, along with the ShortBRED software and documentation, at the ShortBRED website: http://huttenhower.sph.harvard.edu/shortbred. Although the preceding examples have focused on applying ShortBRED to profile antibiotic resistance in genomes and metagenomes, this is only one possible application. Indeed, the same analyses described above can be applied to a wide range of protein families of interest, limited largely by the imagination of the user. To that end, users who produce marker sets with ShortBRED and who would also like to share them with the scientific community are encouraged to submit the markers (along with a relevant citation) for posting on the ShortBRED website.

## Methods

### Creating protein family-specific marker sequences with ShortBRED-Identify

ShortBRED-Identify takes two inputs: (i) a FASTA file of proteins of interest and (ii) a comprehensive catalog of reference protein sequences (as a FASTA file or preformatted BLAST database). The reference database used here was based on version 3.5 of the Integrated Microbial Genomes database [24]. The full version of this database contained 12,607,998 protein coding sequences, which we previously reduced to 4,981,629 representative protein coding sequences proteins by clustering at 80% nucleotide identity [36]. As of this writing, IMG is no longer available for download, and we recommend using UniRef100 or UniRef90 as alternative comprehensive protein reference datasets [37].

ShortBRED-Identify first clusters the proteins of interest at 85% identity using CD-HIT [38, 39] to group them into highly conserved protein families. For each clustered protein family, ShortBRED-Identify first calls MUSCLE [40] to generate a multiple sequence alignment (MSA) for the family, then uses Biopython [41] to generate a consensus sequence for the MSA. If the most common amino acid for a given MSA column occurred in less than 95% of sequences, the corresponding position in the consensus sequence is marked as ambiguous (“X”).

ShortBRED-Identify then uses BLAST [4] to query consensus sequences (i) against one another and (ii) against the reference protein database. The results of these searches are used to identify short segments of each consensus sequence that align with high sequence identity (≥90%) to unrelated proteins in the reference database, or share high identity with a length greater than 80% of minimum marker length with other consensus sequences. (A short sequence is defined as having a length between 80% of the minimum marker length and 15% of a target sequence in the reference database.) Metagenomic reads derived from such segments will be prone to false positive matches across protein families. ShortBRED-Identify thus interprets the BLAST results to find segments of a consensus sequence that participate in a minimal number of such alignments (markers) and then uses these sequences as a basis for more accurate functional profiling. Consensus sequences from different families can share long regions of similarity even after initial clustering at high sequence identity. Because of this, ShortBRED-Identify penalizes high-identity alignments of any length greater than 80% of marker length between pairs of consensus sequences in order to minimize inter-family false positives. ShortBRED does not penalize high-coverage, high-identity alignments between a consensus sequence and a protein from the reference database, as such proteins are likely members of the protein family represented by the consensus.

ShortBRED counts the number of times each amino acid of each consensus sequence appeared in a valid alignment with another protein. These “overlap counts” are then used to identify the most representative segments (markers) for the consensus. For a given consensus sequence, ShortBRED will first try to build as many “True Markers” as possible. A True Marker is a contiguous sequence of amino acids with zero overlap count; i.e. the corresponding peptide was unique among the consensus sequences and non-member reference sequences. If no True Markers are found above a minimum length (with a default of 8 amino acids), ShortBRED next tries to make up to three “Junction Markers” for the consensus sequence. A Junction Marker is a sequence of amino acids that partially overlaps with other consensus sequences or reference sequences, but is not completely overlapped by any single consensus or reference sequence. Note that when mapping reads to marker sequences, ShortBRED-Quantify requires high-identity (≥95%) and high-length (minimum of the marker’s length or 95% of a read length), and hence these partial overlaps will not lead to false positive matches. If it is not possible to build a True Marker or a Junction Marker for a consensus sequence, ShortBRED-Identify will create a single “Quasi Marker” for the consensus, which is a sequence of amino acids above a given minimum length (with a default of 33 amino acids) that has the lowest total adjusted overlap count. The adjusted overlap count is the fourth root of the raw overlap count, and helps to down-weight very short outlier regions with extremely high overlap counts. Protein families with similar Quasi Markers and Junction Markers (≥95% identity) are merged, and then all marker sequences are output as a FASTA file for use by ShortBRED-Quantify.

For Junction Markers and Quasi Markers, ShortBRED also lists the percentage of each marker that overlaps with each other consensus sequence. Each sequence is given a weight, which is defined as its total length in amino acids divided by the sum of that value, and all overlapping amino acids from other reference or consensus sequences. The weight is printed in the FASTA header, along with other highly overlapping consensus sequences from the input database. An additional text file lists the overlapping regions from the consensus sequences and the reference database.

This ShortBRED-Identify process requires ~100 CPU-hours to complete given a set of proteins of interest which cluster to ~1,000 protein families. The bottleneck in this process is the BLAST-based search of the protein family consensus sequences against the comprehensive reference database. Notably, this process is highly parallelizable, as each consensus sequence can be searched independently of the others. By allowing ShortBRED-Identify to use multiple cores during the search process, the actual run-time can be reduced considerably. Once the initial BLAST results have been generated, new markers can be generated in a few minutes provided that the initial clustering identity and consensus thresholds are not changed. Precomputed markers for the antibiotic resistance proteins (ARDB) [13] and virulence factors (VFDB) [14] are available for download at http://huttenhower.sph.harvard.edu/shortbred. Notably, the ShortBRED-Identify process needs to be applied only once to produce a set of markers, which can then be used repeatedly to profile metagenomic datasets using ShortBRED-Quantify.

### Profiling protein family metagenomic abundance with ShortBRED-Quantify

After markers have been created for each protein family, the user can call ShortBRED-Quantify to profile the relative abundance of these families in a whole metagenomic shotgun (WMS) sequencing sample. ShortBRED-Quantify calls USEARCH [5] to find the best matching marker for each nucleotide read. USEARCH specializes in fast search for high-identity matches, which fits with ShortBRED’s objective of profiling metagenomic samples quickly with high specificity.

By default, ShortBRED-Quantify will record a hit to a marker if the resulting alignment has at least 95% identity, and is at least as long as the minimum of (i) the marker length or (ii) 95% of the read length. For each marker, ShortBRED-Quantify computes an adjusted marker length, which takes into account how much of the marker is available to participate in a hit meeting our length and percent identity requirements. When a marker of length L is longer than the average read length (R), a read from the corresponding gene anywhere in the region from 5% downstream of the marker to 5% upstream of the marker is allowed to align to the marker. Therefore, the adjusted marker length (L’) is:

$$L^{\prime }=L-0.9R+1$$

When the marker is shorter than the expected read length (L<R), the we require the entire marker to align to the read. Thus, the adjusted marker length is:

$$L^{\prime }=R-L-1$$

ShortBRED then normalizes the number of raw USEARCH hits to a marker (H) to produce a normalized count (C), adjusting for average read length, marker length, and sequencing depth (number of reads in the sample, N):

$$C=\frac{H}{\left(\frac{L^{\prime }}{10^{3}}\right)\left(\frac{N}{10^{6}}\right)}=\frac{H}{L^{\prime }N}\times 10^{9}$$

The normalized count is in units of RPKMs (reads per kilobase of reference sequence per million sample reads). For protein families characterized by multiple markers, a normalized count is first computed for each marker separately and then the median of these values is taken to represent the protein family; this procedure adds robustness to variation in sequencing depth across the markers. Finally, ShortBRED-Quantify outputs these normalized counts as a relative abundance table for the protein families of interest.

### Creation of synthetic spiked metagenomes

We used GemSim [42] to create synthetic metagenomes containing five million 100 nucleotide-long reads, designed to mimic a typical WMS-sequencing run on an Illumina HiSeq instrument (Illumina, San Diego, CA). Reads were drawn from twenty bacterial genomes obtained from the KEGG database [43, 44]. We used USEARCH [5] to identify and exclude from these genomes any naturally-occurring antibiotics resistance genes and virulence factors (defined as a sequence matching a gene from the ARDB or VFDB with >90% identity). This ensured that the only ARDB and VFDB sequences in our synthetic metagenomes were those that had been artificially spiked in for the purposes of evaluating ShortBRED. Each bacterial genome was assigned an abundance value drawn from a log-normal distribution with unit mean and standard deviation.

We created six Illumina-like synthetic metagenomes with material spiked in from the ARDB and VFDB sequence datasets. Three metagenomes were made for each dataset, with 150, 500, and 1,000 genes from the corresponding protein dataset spiked among the genomic reads at 5%, 10%, and 25% relative abundance. Two additional sets of Illumina-like synthetic metagenomes were created with 3% and 5% of the amino acid content of the sequences mutated based on relative amino acid mutability and transition probabilities from the BLOSUM62 table. An additional set of six metagenomes were created using the same procedure but based on a simulated 454 sequencing instrument (454 Life Sciences, Branford, CT); these samples contained only 155,890 reads each, consistent with the lower sequencing depth of the 454 platform. We used 164 nucleotide sequences corresponding to ARDB protein sequences as a base for the ARDB metagenomes and 2,296 VFDB nucleotide sequences as a base for VFDB metagenomes. Nucleotide sequences were not always provided for ARDB proteins; in these cases, we used the EMBOSS program *backtranseq* [45] to create nucleotide sequences that were compatible with the available amino acid sequences.

Code for creating the synthetic metagenomes can be found at http://bitbucket.org/biobakery/shortbred_doit.

### Application of ShortBRED to human gut metagenomes

We applied ShortBRED to profile antibiotic resistance (AR) in the human gut microbiome. We first produced a set of new AR marker sequences by applying ShortBRED-Identify to a combination of (i) a curated version of the ARDB which we obtained by deleting sequences no longer stored at NCBI and (ii) a set of known antibiotic resistance genes obtained from resistant bacterial libraries. We then used ShortBRED-Quantify to profile the relative abundance of corresponding AR protein families across 552 gut metagenomes: 82 from U.S. adults sampled during the Human Microbiome Project (HMP) [10], 363 from Chinese adults with and without diabetes [11], and 107 individuals from Malawi, Venezuela, and the U.S. [12]. We used the first-visit samples from multi-visit HMP subjects to avoid redundancy. For 454-based samples characterized by sub-optimal sequencing depth, we mapped reads to full-length centroid sequences to avoid compromising sensitivity.

### Application of ShortBRED to bacterial isolate genomes

ShortBRED can be applied to identify protein families in a bacterial isolate genome given a corresponding set of ShortBRED markers for those families. To do so, ShortBRED first creates a USEARCH database for the genome and then searches the markers against that database (allowing for multiple hits per marker query). For protein families characterized by more than one marker sequence, ShortBRED requires that a critical fraction of the markers map to a gene in the genome before assigning it to that protein family. The default value for this cutoff is 10% [i.e. 1 in 10 markers], but it can be tuned to be more conservative.

## Supporting Information

S1 Fig Training/testing of ShortBRED’s default parameters–ARDB AUC. Values reflect area under the ROC curve (AUC) as minimum marker length and initial clustering ID are varied. This analysis was based on the 5%-spiked synthetic metagenomes containing ARDB sequences.
(PDF)

S2 Fig Training/testing of ShortBRED’s default parameters–VFDB AUC. Values reflect area under the ROC curve (AUC) as minimum marker length and initial clustering ID are varied. This analysis was based on the 5%-spiked synthetic metagenomes containing VFDB sequences.
(PDF)

S3 Fig Training/testing of ShortBRED’s default parameters–ARDB specificity. Values reflect specificity as minimum marker length and initial clustering ID are varied. This analysis was based on the 5%-spiked synthetic metagenomes containing ARDB sequences.
(PDF)

S4 Fig Training/testing of ShortBRED’s default parameters–VFDB specificity. Values reflect specificity as minimum marker length and initial clustering ID are varied. This analysis was based on the 5%-spiked synthetic metagenomes containing VFDB sequences.
(PDF)

S5 Fig Training/testing of ShortBRED’s default parameters–ARDB sensitivity. Values reflect sensitivity as minimum marker length and initial clustering ID are varied. This analysis was based on the 5%-spiked synthetic metagenomes containing ARDB sequences.
(PDF)

S6 Fig Training/testing of ShortBRED’s default parameters–VFDB sensitivity. Values reflect sensitivity as minimum marker length and initial clustering ID are varied. This analysis was based on the 5%-spiked synthetic metagenomes containing VF sequences.
(PDF)

S7 Fig Training/testing of ShortBRED’s default parameters–ARDB Spearman correlation. Values reflect Spearman correlation between estimated abundances and true abundances as minimum marker length and initial clustering ID are varied. This analysis was based on the 5%-spiked synthetic metagenomes containing ARDB sequences.
(PDF)

S8 Fig Training/testing of ShortBRED’s default parameters–VFDB Spearman correlation. Values reflect Spearman correlation between estimated abundances and true abundances as minimum marker length and initial clustering ID are varied. This analysis was based on the 5%-spiked synthetic metagenomes containing VFDB sequences.
(PDF)

S9 Fig Accuracy of ShortBRED and centroid-based mapping in applications involving 454 sequencing data. (**A**) and (**B**) report the sensitivity and specificity of the two methods for mapping reads to their correct families on six synthetic 454 metagenomes, spiked with 5%, 10%, and 25% of their material from the ARDB (panel A) and VFDB (panel B). (**C**) and (**D**) display scatterplots of protein family “predicted by mapping”, the abundance values calculated by ShortBRED and the centroids, vs. “expected from gold standard”, the abundance values of the protein families in the 10% synthetic metagenome. This figure is an analog of Fig 2 from the main text.
(PDF)

S10 Fig Prevalence of all antibiotic resistance classes across bacterial isolate genomes. Phylogenetic tree of bacterial genomes from IMG [24] overlaid with presence/absence of ShortBRED antibiotic resistance protein families. The outermost ring indicates the share of genes in the species’ genome that mapped to any of the AR protein families.
(PNG)

S1 Table Performance of ShortBRED and centroid method across synthetic metagenomes. This table displays measures of the performance of ShortBRED and centroids to profile synthetic metagenomes. The results for ShortBRED using its default settings on the six synthetic metagenomes are in bold. Illumina_05,Illumina_10, and Illumina_25 represent synthetic Illumina metagenomes with reads from input dataset sequences comprising 5%, 10%, and 25% of the metagenome. Illumina-mutated-3pct and Illumina-mutated-5pct metagenomes had 3% and 5% of their amino acid content mutated before being incorporated into the metagenome. Centroids were also obtained by clustering at 85% identity. Any result mapping to centroid with length ≥30 and ID ≥95% was considered a “match”.
(XLSX)

S2 Table False positive yeast hits detected by centroids in synthetic bacterial metagenome. These are yeast centroids which had false positive matches to reads from a synthetic bacterial metagenome at high identity (≥95%) and long length ≥30 amino acids. Source: Kyoto Encyclopedia of Genes and Genomes (KEGG) (Kanehisa et al, 2000 and 2012)
(XLSX)

S3 Table ShortBRED counts (RPKM) by AR family for each microbiome sample. Each row of this table represents a family of antibiotic resistance (AR) proteins and each column corresponds to a sample in our dataset. The abundance of the AR family in the sample is given in RPKM.
(XLSX)

S4 Table Summary statistics of ShortBRED counts for AR families in metagenomes. Each row represents a family of antibiotic resistance (AR) proteins. Column B lists the corresponding AR class. (The classes are groups of AR families.) The columns provide summary statistics of the ShortBRED RPKM counts for each family. Sample statistics for columns C through I are based on the entire sample of all three datasets. Summary statistics are also provided for the individual three datasets. Please note that the Yatsunenko et al dataset results were based on running ShortBRED using the centroids, as opposed to the markers from ShortBRED-Identify. This is because this particular dataset has shallower coverage than the Illumina datasets (HMP and T2D).
(XLSX)

S5 Table Summary statistics of AR ORF counts in bacterial genomes by AR class. Each row in this table represents summary statistics for the abundance of a particular AR class in a genus of bacteria. We used 3,305 bacterial genomes obtained from the Integrated Microbial Genomes database. In "annotated genome" mode, ShortBRED takes a fasta file of open reading frames from the genome, calls USEARCH to build a database, and then checks the markers against the database for hits.
(XLSX)

S6 Table ShortBRED count for AR class minus count for "genome by median gene" method. This table presents the differences between counting antibiotic resistance (AR) abundance in microbiome samples using ShortBRED, and inferring AR abundance in the same samples based on the abundance of bacteria in the microbiome multiplied by the median count of each AR class found in available bacterial genomes. Each row is a class, each column is sample from the HMP and the value in each cell equals [ShortBRED Count in RPKM—Sum(Bacterial Genome's Abundance in Sample * Median ShortBRED AR Count Across Copies of Bacterial Genome in our 3,305 isolates from IMG.]
(XLSX)
