# Supplementary figures and images for: High-Specificity Targeted Functional Profiling in Microbial Communities with ShortBRED
Source: PLoS Comput Biol. 2015 Dec 18;11(12):e1004557. doi: 10.1371/journal.pcbi.1004557 (PMC4684307; doi:10.1371/journal.pcbi.1004557)

# ShortBRED AUC on the ARDB, Matching at 95% Identity

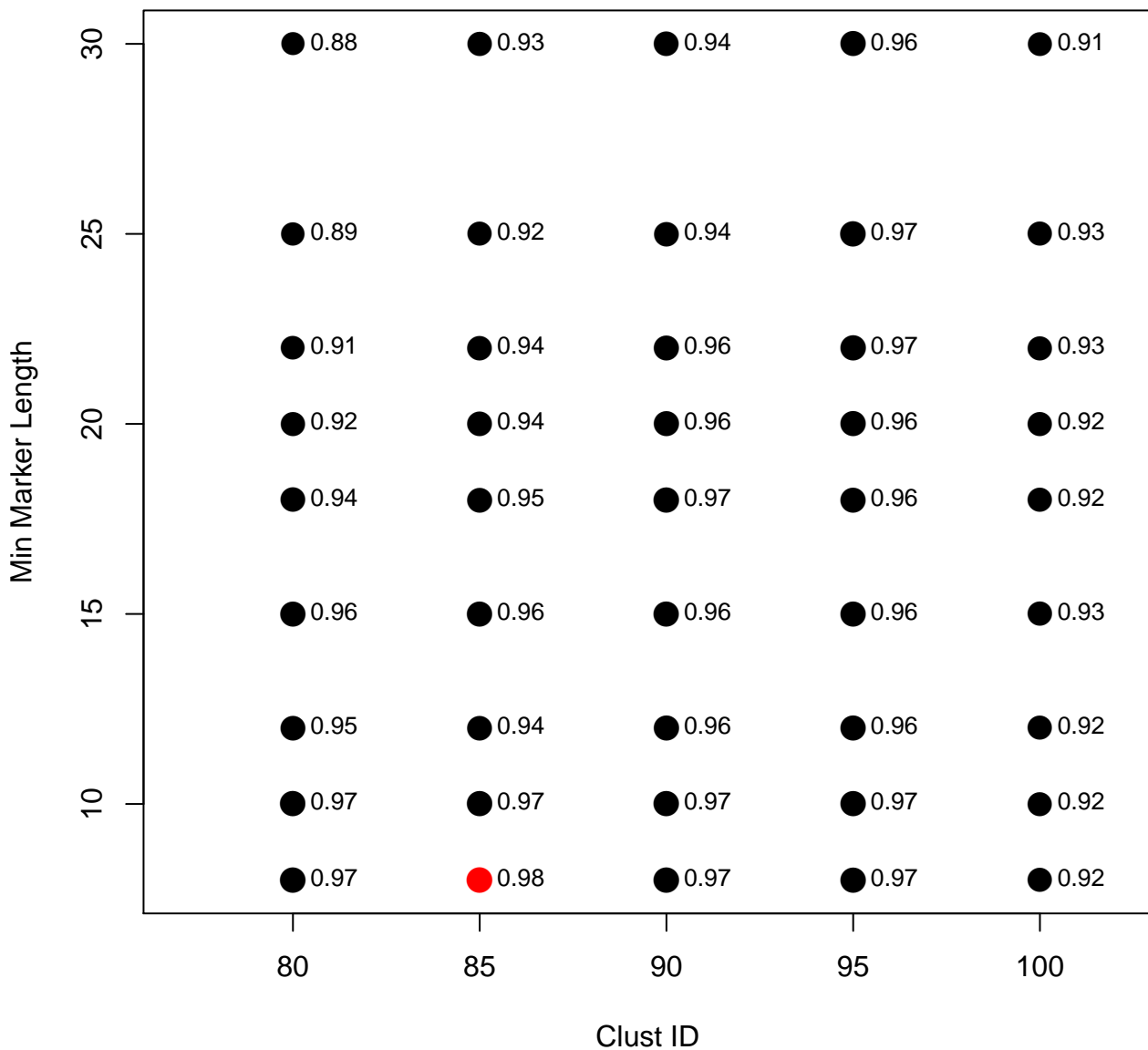

Supplement: S1 Fig — Values reflect area under the ROC curve (AUC) as minimum marker length and initial clustering ID are varied. This analysis was based on the 5%-spiked synthetic metagenomes containing ARDB sequences. (PDF) [file pcbi.1004557.s001.pdf]

## ShortBRED AUC on the VF, Matching at 95% Identity

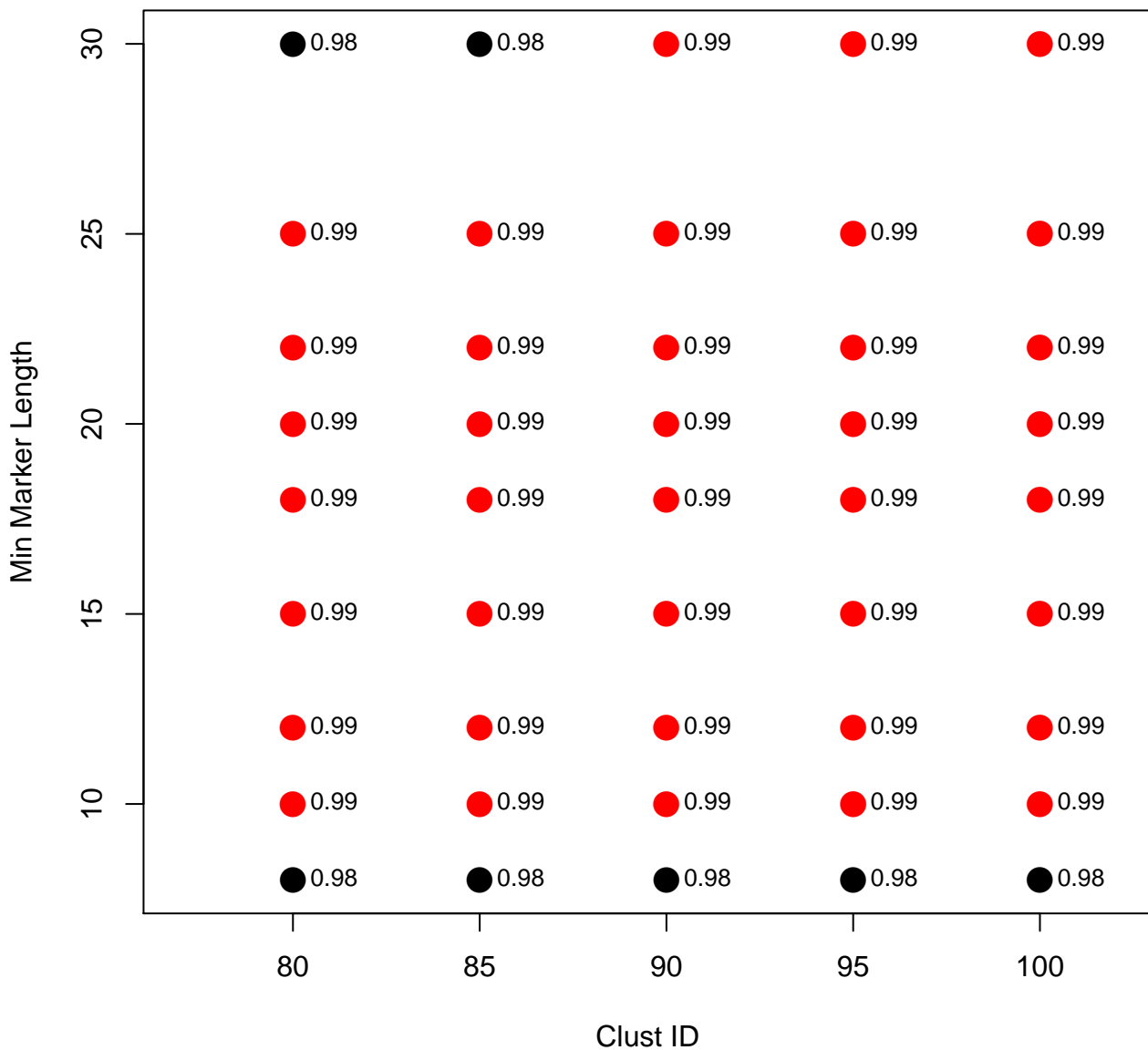

Supplement: S2 Fig — Values reflect area under the ROC curve (AUC) as minimum marker length and initial clustering ID are varied. This analysis was based on the 5%-spiked synthetic metagenomes containing VFDB sequences. (PDF) [file pcbi.1004557.s002.pdf]

# ShortBRED Specificity on the ARDB, Matching at 95% Identity

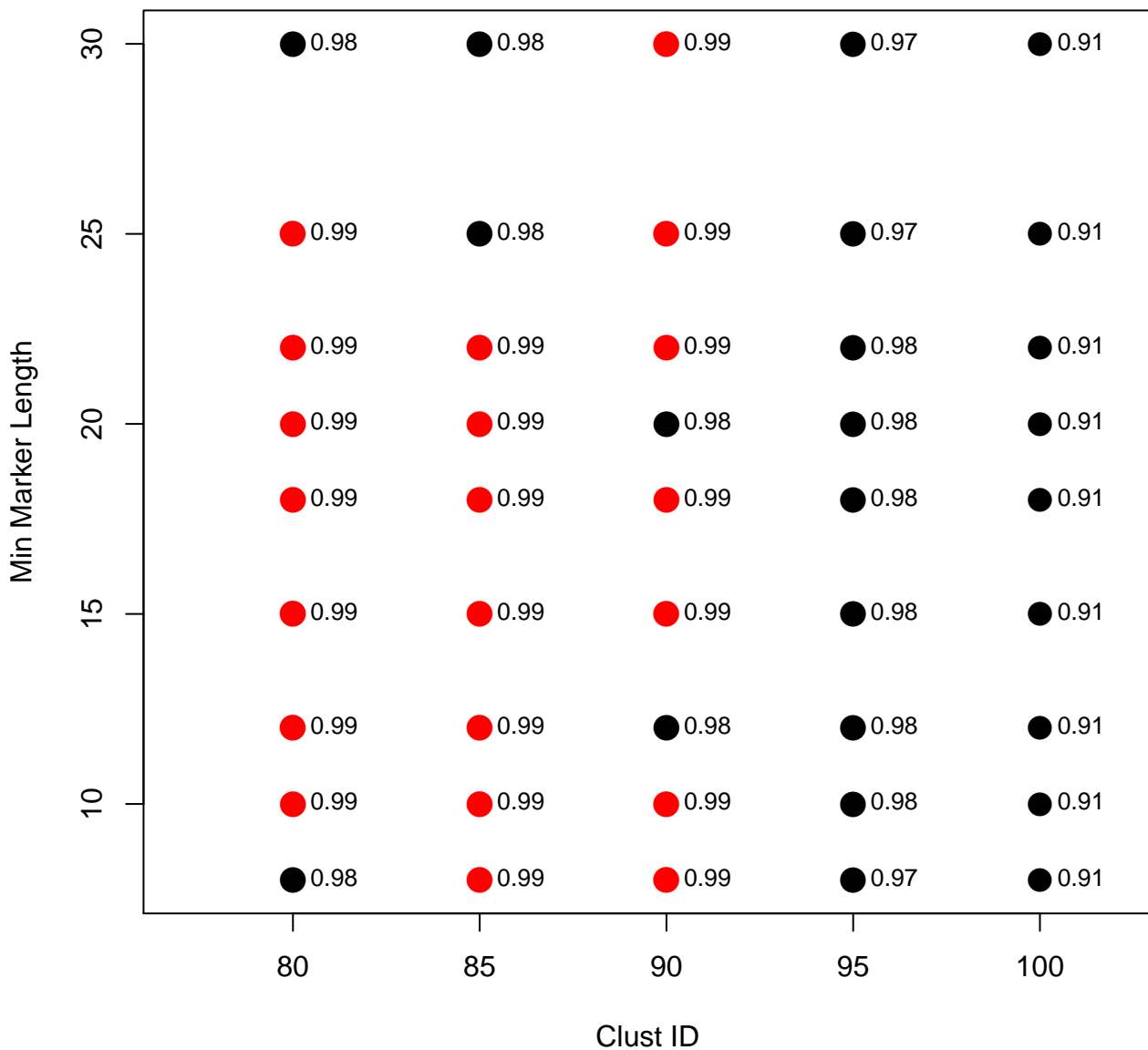

Supplement: S3 Fig — Values reflect specificity as minimum marker length and initial clustering ID are varied. This analysis was based on the 5%-spiked synthetic metagenomes containing ARDB sequences. (PDF) [file pcbi.1004557.s003.pdf]

# ShortBRED Specificity on the VF, Matching at 95% Identity

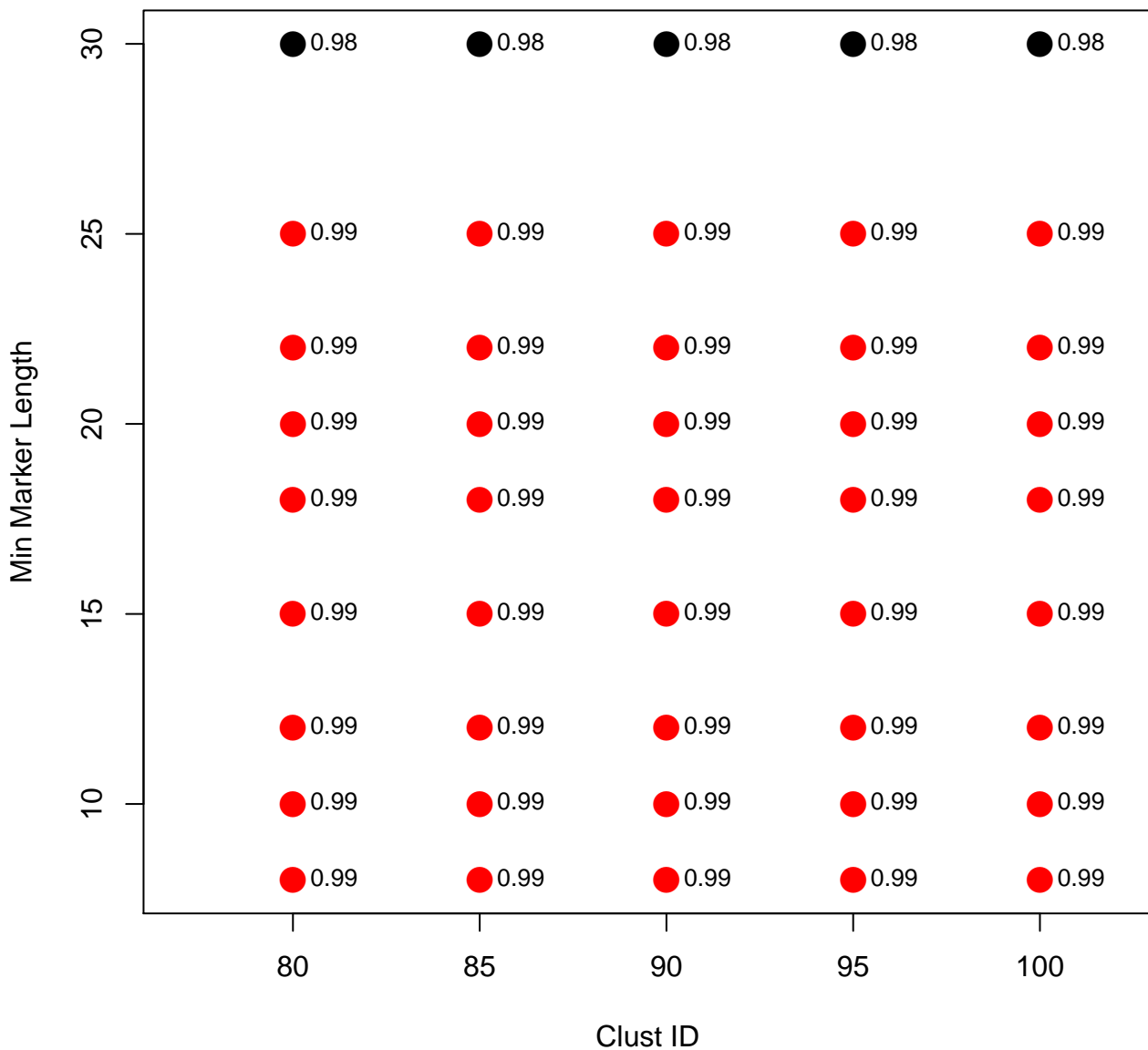

Supplement: S4 Fig — Values reflect specificity as minimum marker length and initial clustering ID are varied. This analysis was based on the 5%-spiked synthetic metagenomes containing VFDB sequences. (PDF) [file pcbi.1004557.s004.pdf]

## ShortBRED Sensitivity on the ARDB, Matching at 95% Identity

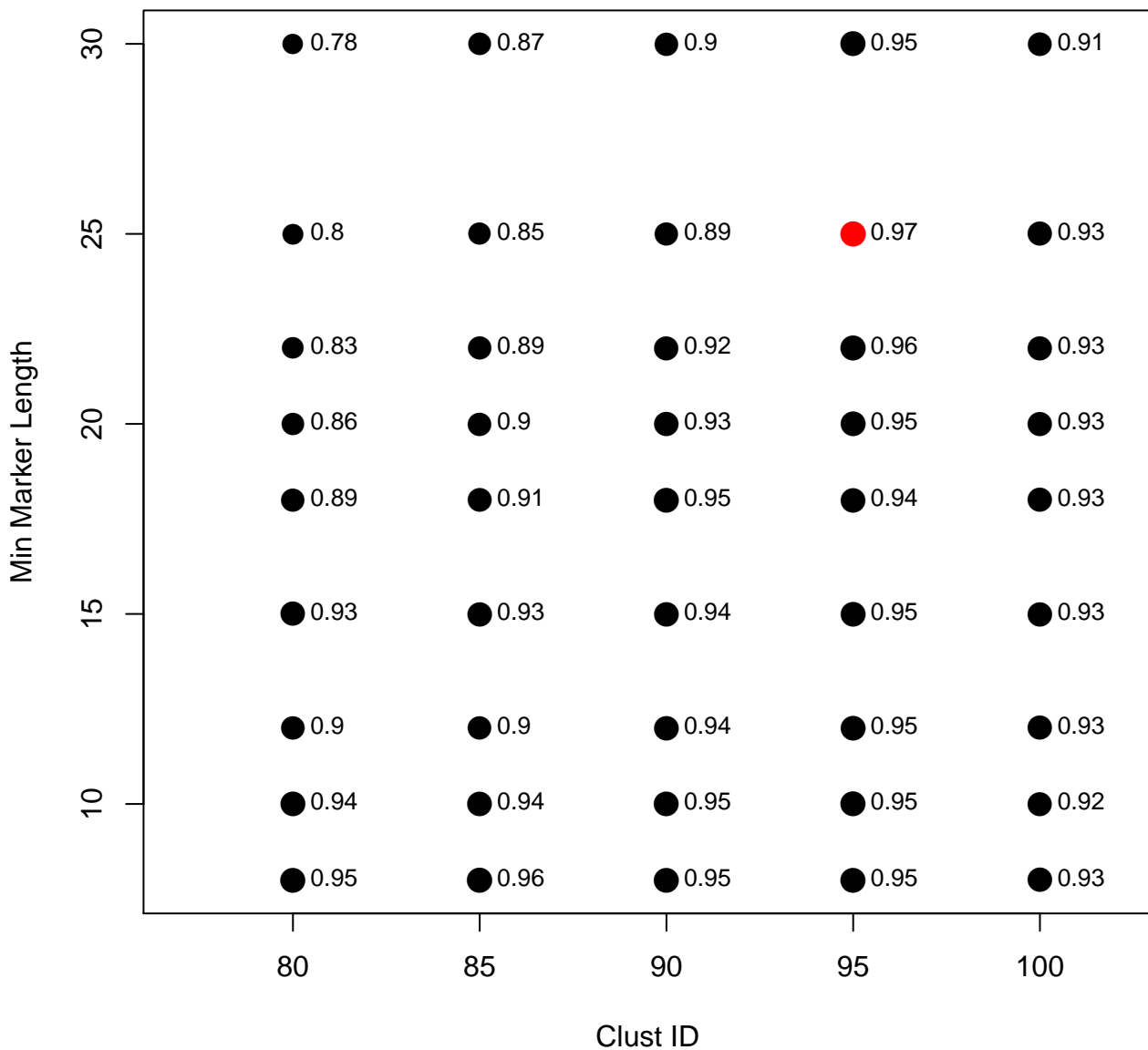

Supplement: S5 Fig — Values reflect sensitivity as minimum marker length and initial clustering ID are varied. This analysis was based on the 5%-spiked synthetic metagenomes containing ARDB sequences. (PDF) [file pcbi.1004557.s005.pdf]

# ShortBRED Sensitivity on the VF, Matching at 95% Identity

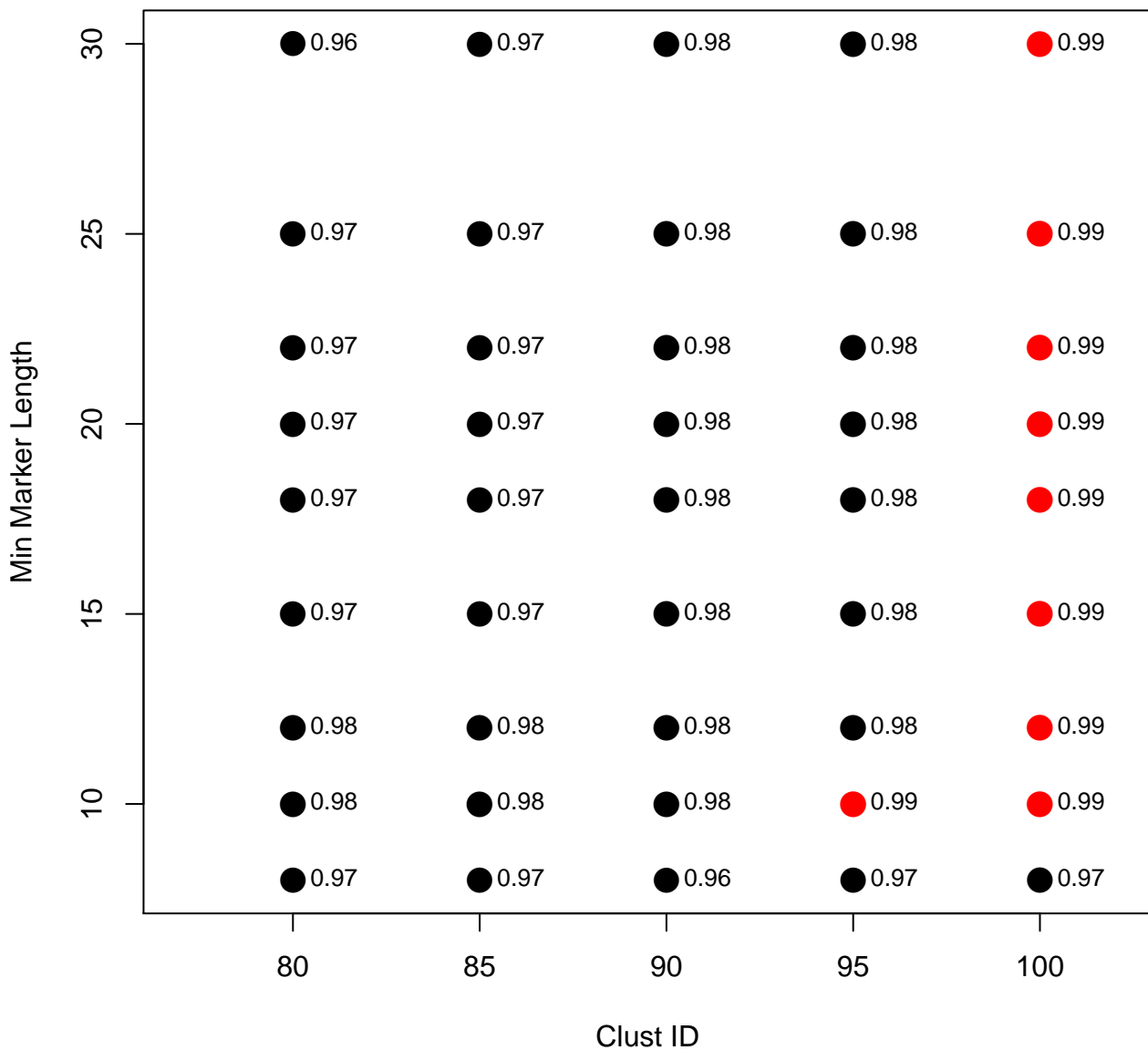

Supplement: S6 Fig — Values reflect sensitivity as minimum marker length and initial clustering ID are varied. This analysis was based on the 5%-spiked synthetic metagenomes containing VF sequences. (PDF) [file pcbi.1004557.s006.pdf]

# ShortBRED Spearman Correlation on the ARDB, Matching at 95% Identity

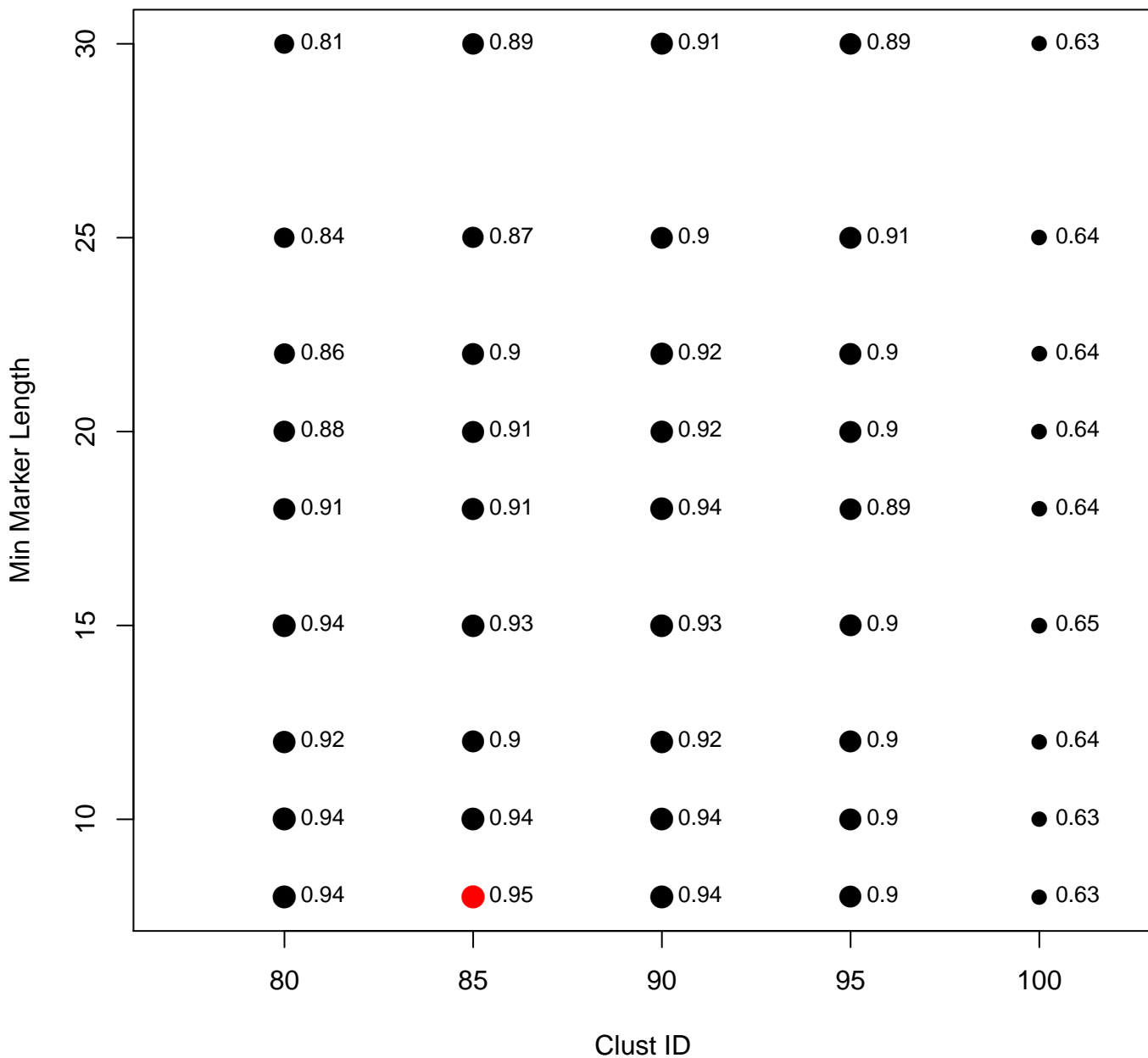

Supplement: S7 Fig — Values reflect Spearman correlation between estimated abundances and true abundances as minimum marker length and initial clustering ID are varied. This analysis was based on the 5%-spiked synthetic metagenomes containing ARDB sequences. (PDF) [file pcbi.1004557.s007.pdf]

# ShortBRED Spearman Correlation on the VF, Matching at 95% Identity

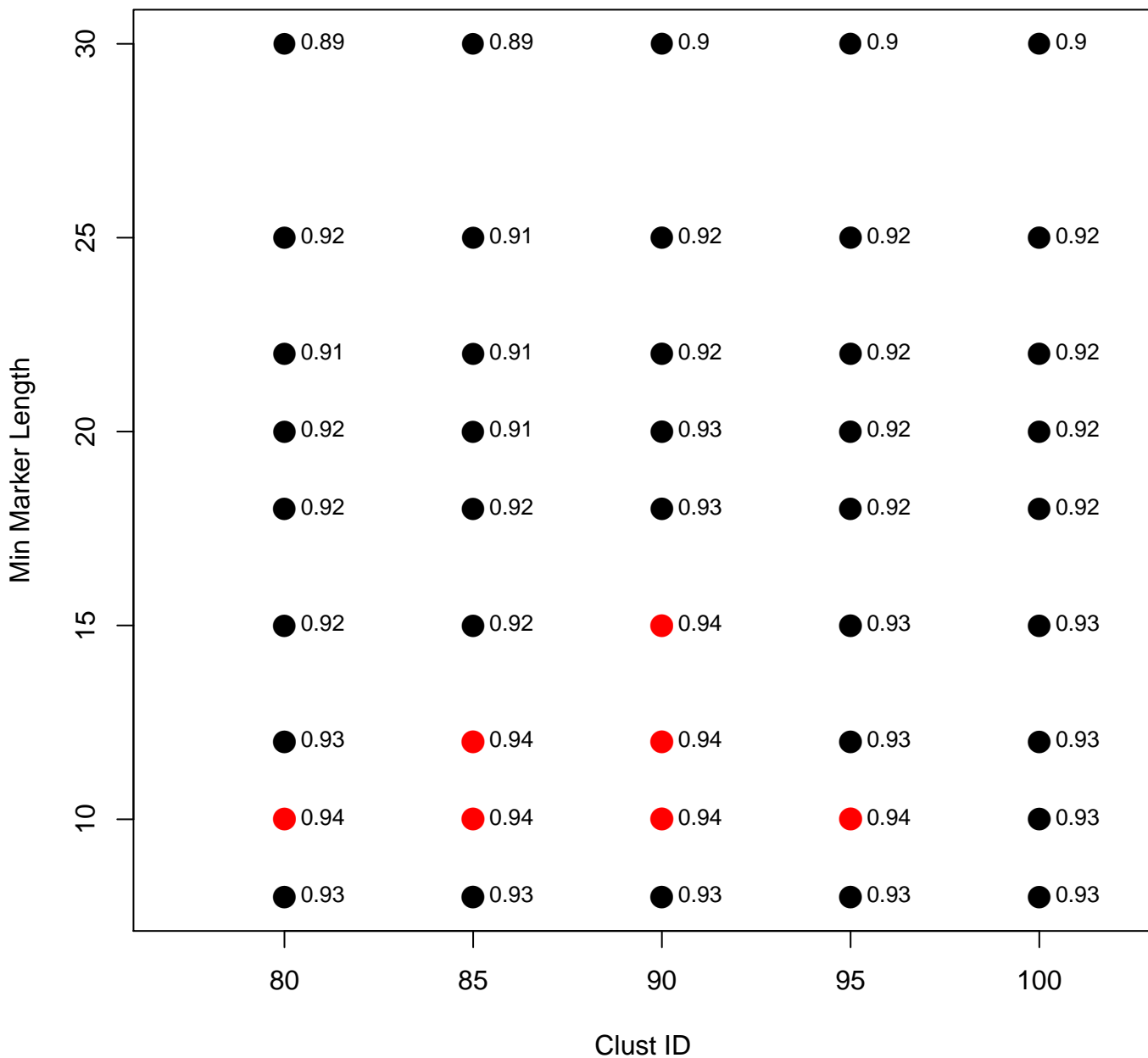

Supplement: S8 Fig — Values reflect Spearman correlation between estimated abundances and true abundances as minimum marker length and initial clustering ID are varied. This analysis was based on the 5%-spiked synthetic metagenomes containing VFDB sequences. (PDF) [file pcbi.1004557.s008.pdf]

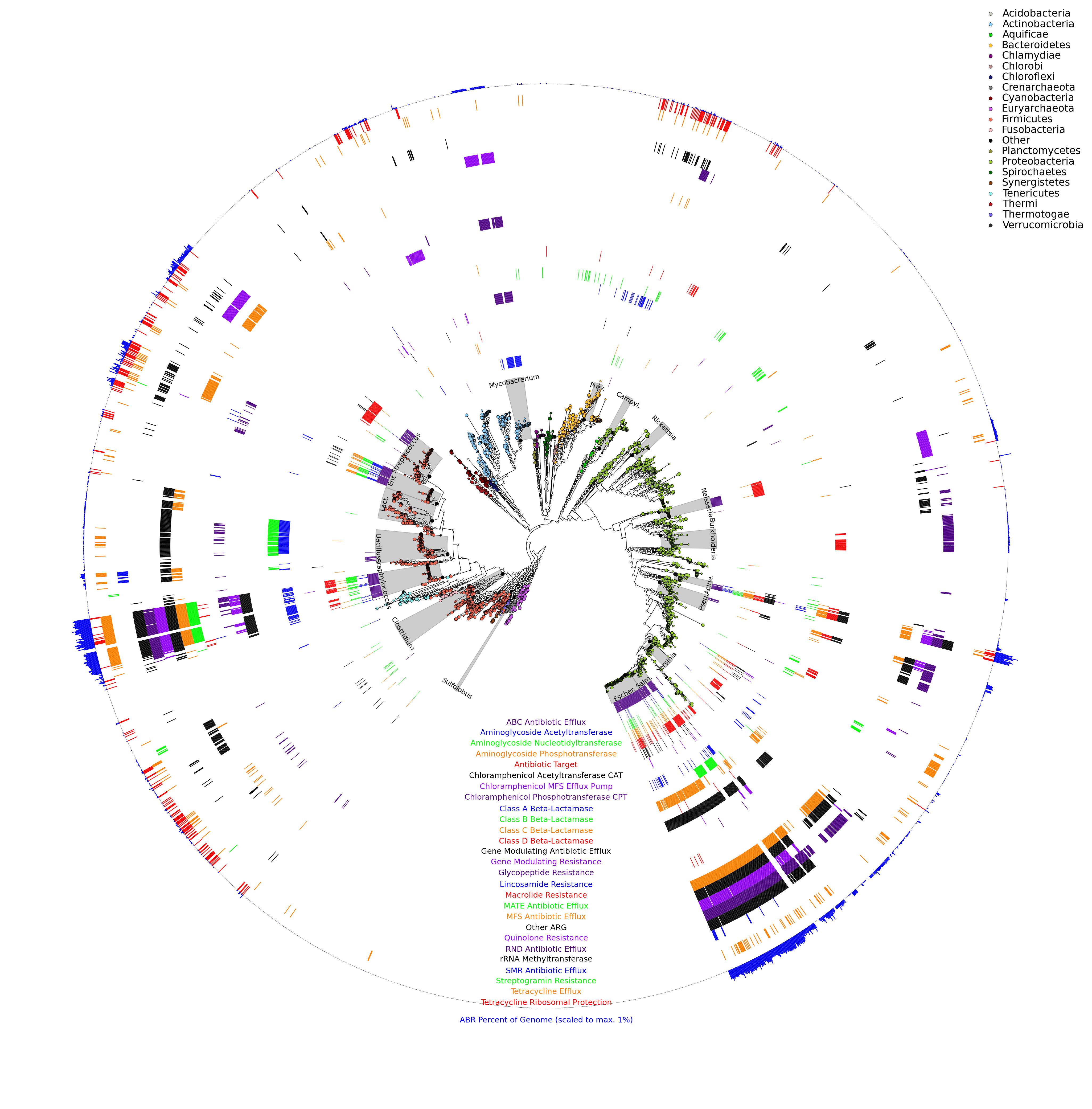

Supplement: S10 Fig — Phylogenetic tree of bacterial genomes from IMG [24] overlaid with presence/absence of ShortBRED antibiotic resistance protein families. The outermost ring indicates the share of genes in the species’ genome that mapped to any of the AR protein families. (PNG) [file pcbi.1004557.s010.png]
