# Supplementary material for: High-Specificity Targeted Functional Profiling in Microbial Communities with ShortBRED
Source: PLoS Comput Biol. 2015 Dec 18;11(12):e1004557. doi: 10.1371/journal.pcbi.1004557 (PMC4684307; doi:10.1371/journal.pcbi.1004557)
Supplement: S9 Fig — (A) and (B) report the sensitivity and specificity of the two methods for mapping reads to their correct families on six synthetic 454 metagenomes, spiked with 5%, 10%, and 25% of their material from the ARDB (panel A) and VFDB (panel B). (C) and (D) display scatterplots of protein family “predicted by mapping”, the abundance values calculated by ShortBRED and the centroids, vs. “expected from gold standard”, the abundance values of the protein families in the 10% synthetic metagenome. This figure is an analog of Fig 2 from the main text. (PDF) [file pcbi.1004557.s009.pdf]

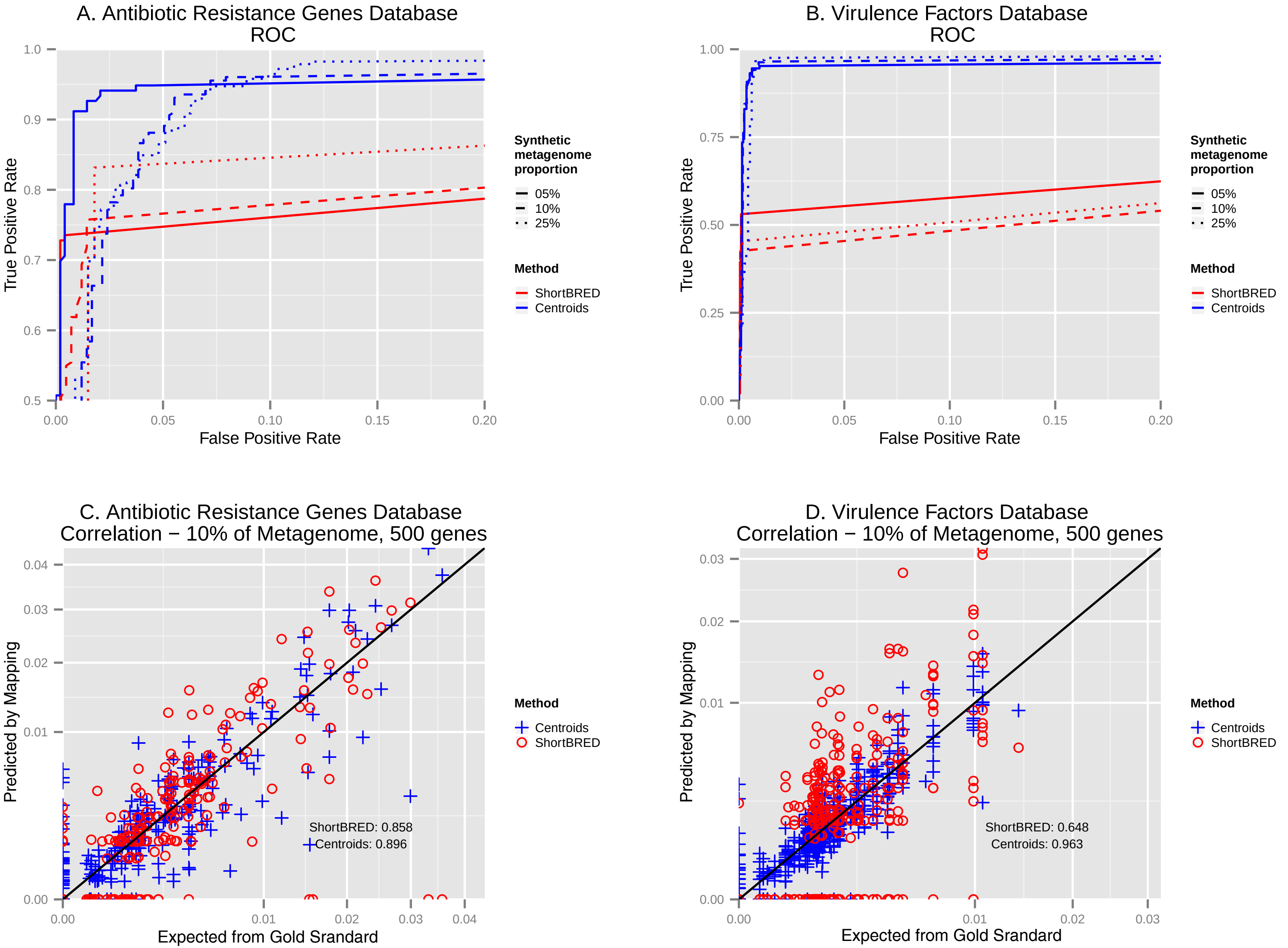

A. Antibiotic Resistance Genes Database
ROC
True Positive Rate
False Positive Rate
Synthetic metagenome proportion
05%
10%
25%
Method
ShortBRED
Centroids
B. Virulence Factors Database
ROC
True Positive Rate
False Positive Rate
Synthetic metagenome proportion
05%
10%
25%
Method
ShortBRED
Centroids
C. Antibiotic Resistance Genes Database
Correlation − 10% of Metagenome, 500 genes
Predicted by Mapping
Expected from Gold Srandard
ShortBRED: 0.858
Centroids: 0.896
Method
Centroids
ShortBRED
D. Virulence Factors Database
Correlation − 10% of Metagenome, 500 genes
Predicted by Mapping
Expected from Gold Srandard
ShortBRED: 0.648
Centroids: 0.963
Method
Centroids
ShortBRED
